# Supplementary material for: Actin polymerisation and crosslinking drive left-right asymmetry in single cell and cell collectives
Source: Nat Commun. 2023 Feb 11;14:776. doi: 10.1038/s41467-023-35918-1 (PMC9922260; doi:10.1038/s41467-023-35918-1)
Supplement: Supplementary file 1 — Supplementary Information [file 41467_2023_35918_MOESM1_ESM.pdf]

Supplementary Information for

Actin polymerisation and crosslinking drive left-right asymmetry in single cell and cell collectives

Tee *et al.*

This PDF file includes:

Supplementary Figs 1 to 13

Supplementary Tables 1 to 4

Other Supplementary Materials for this manuscript include the following:

Supplementary Movies 1 to 8

Description of additional supplementary files

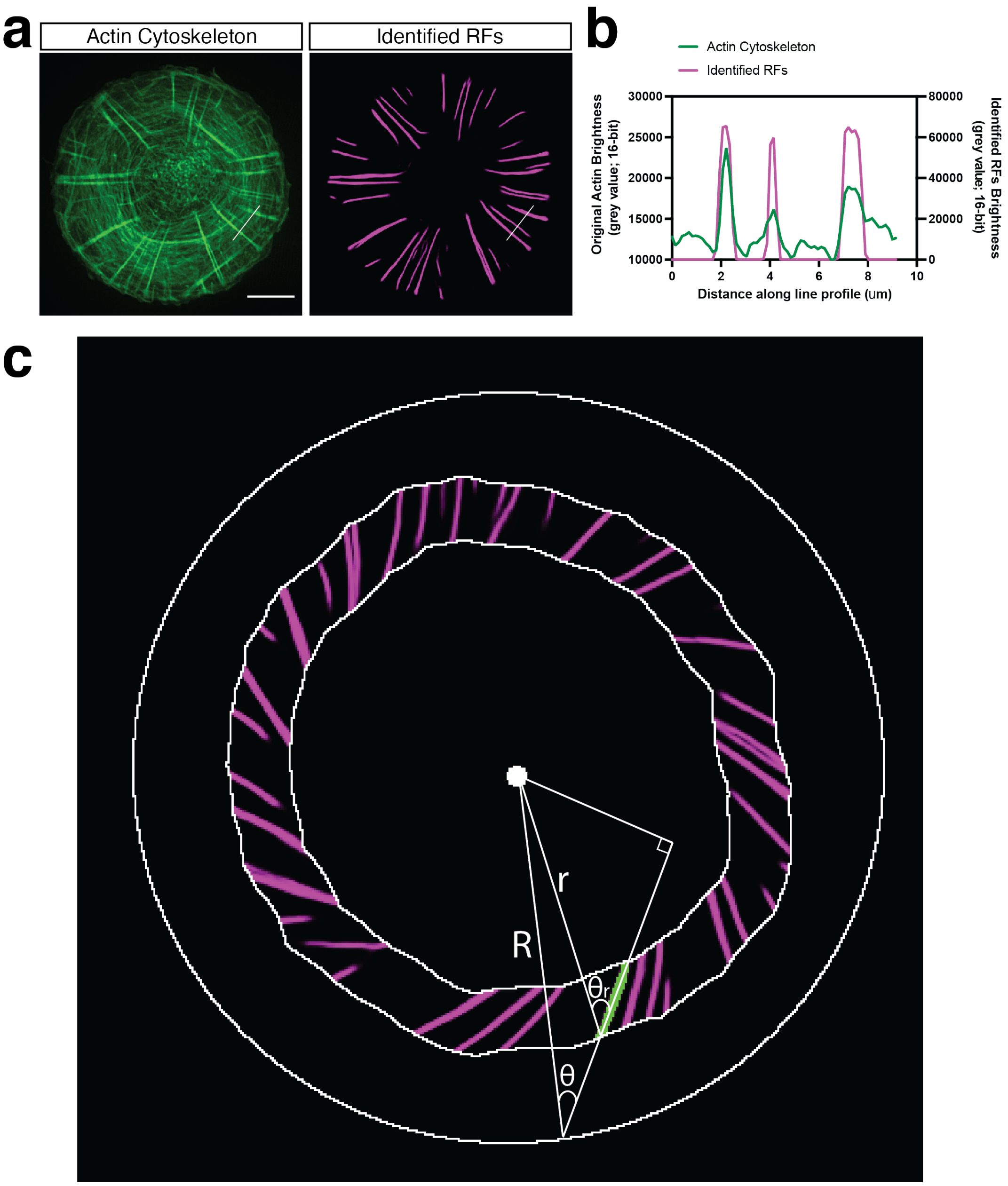

$$\theta = \arcsin\left(\frac{r \sin \theta_r}{R}\right)$$

Supplementary Figure 1. Calculation of radial fibre tilt.

a A fluorescence image of phalloidin-labelled actin cytoskeleton and its corresponding identified radial fibres (RFs) by deep-learning procedure. Scale bar, 10 µm.

b Line profile of brightness values (in 16-bit grey scale) of actin cytoskeleton in original image (green) and identified RFs (magenta) along the white line shown in (**a**)**.** The three peaks along actin cytoskeleton line profile represent RFs of varied brightness and width. Note that three corresponding peaks along magenta line have similar brightness.

c The tilts of all radial fibre segments in concentric belts (annuli) located at given distance from the cell edge were measured for each circular cell. The radial fibre segments in the 6–10 μm annulus is shown here. The tilt θ of a single radial fibre segment (highlighted in green) was calculated according to the formula below. $\vec{R}$ connects the cell centroid and intersection of the continuation of the radial fibre segment with the edge of the cell. $\vec{r}$ connects the cell centroid and intersection of radial fibre with outer edge of the annulus. θ and θ_r are the angles between the radial fibre segment and $\vec{R}$ and $\vec{r}$ respectively.

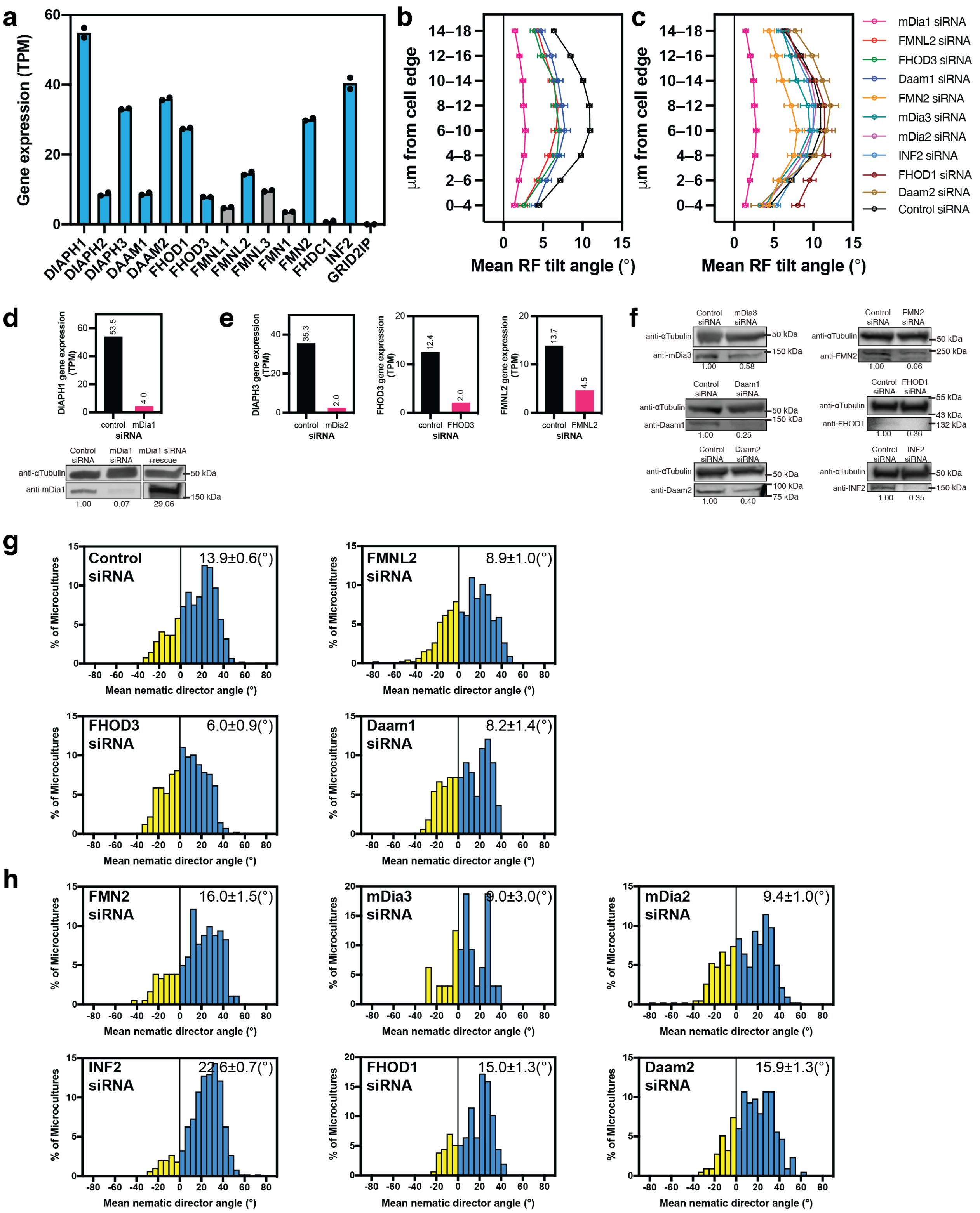

Supplementary Figure 2. Effects of knockdown of formin family proteins on left-right asymmetry of actin organisation in individual cells and chiral cell alignment in microcultures.

a Transcriptome profiling of gene expression levels (transcripts per million; TPM) of the 15 mammalian formin members identified by RNA-sequencing (RNA-seq) (mean values of n= 2 experiments) in human fibroblasts. Cyan bars represent formins examined in this study.
b,**c** Average values of radial fibre (RF) tilts (mean±SEM) as a function of the distance of annuli from the cell edge for two groups of formin family members, knockdown of which either reduced (**b**) or did not apparently affect (**c**) actin cytoskeleton chirality. Classification of formins into group (**b**) and (**c**) is based on statistical analysis shown in Supplementary Table 1, lines 124–133. Graphs corresponding to mDia1 (magenta) and control (black) siRNAs are presented in both (**b**) and (**c**). Mean±SEM of the distribution of average RF tilt in the 6–10 μm annulus of the various knockdowns can be found in Supplementary Table 2.
d siRNA knockdown of mDia1 (DIAPH1) in fibroblasts as verified by RNA-profiling (top) and western blot (bottom). Rescue of mDia1 knockdown cells by co-transfection with mDia1 full-length plasmid is shown in lane 3 of western blot.
e,**f** Gene expression levels (**e**) and western blots (**f**) showing individual formin protein levels in scrambled control siRNA and formin specific siRNAs -treated cells. Fold change in protein expression levels normalised to loading control (α-Tubulin) between control siRNA-treated cell (expression taken as 1.00) and cells treated with corresponding siRNAs are indicated at the bottom of each blot in (**d**) and (**f**).
g,h Quantification of chiral alignment of cells with formin protein knockdowns in microcultures as characterised by mean nematic directors angle. Mean±SEM values are indicated at the top right corner of each histogram. Negative and positive values are coloured in yellow and cyan respectively.
Sample sizes (n) for (**b**), (**c**), (**g**) and (**h**) can be found in Supplementary Table 2. See uncropped blots in Source Data. For statistical analysis, see Supplementary Table 1, lines 124–166.

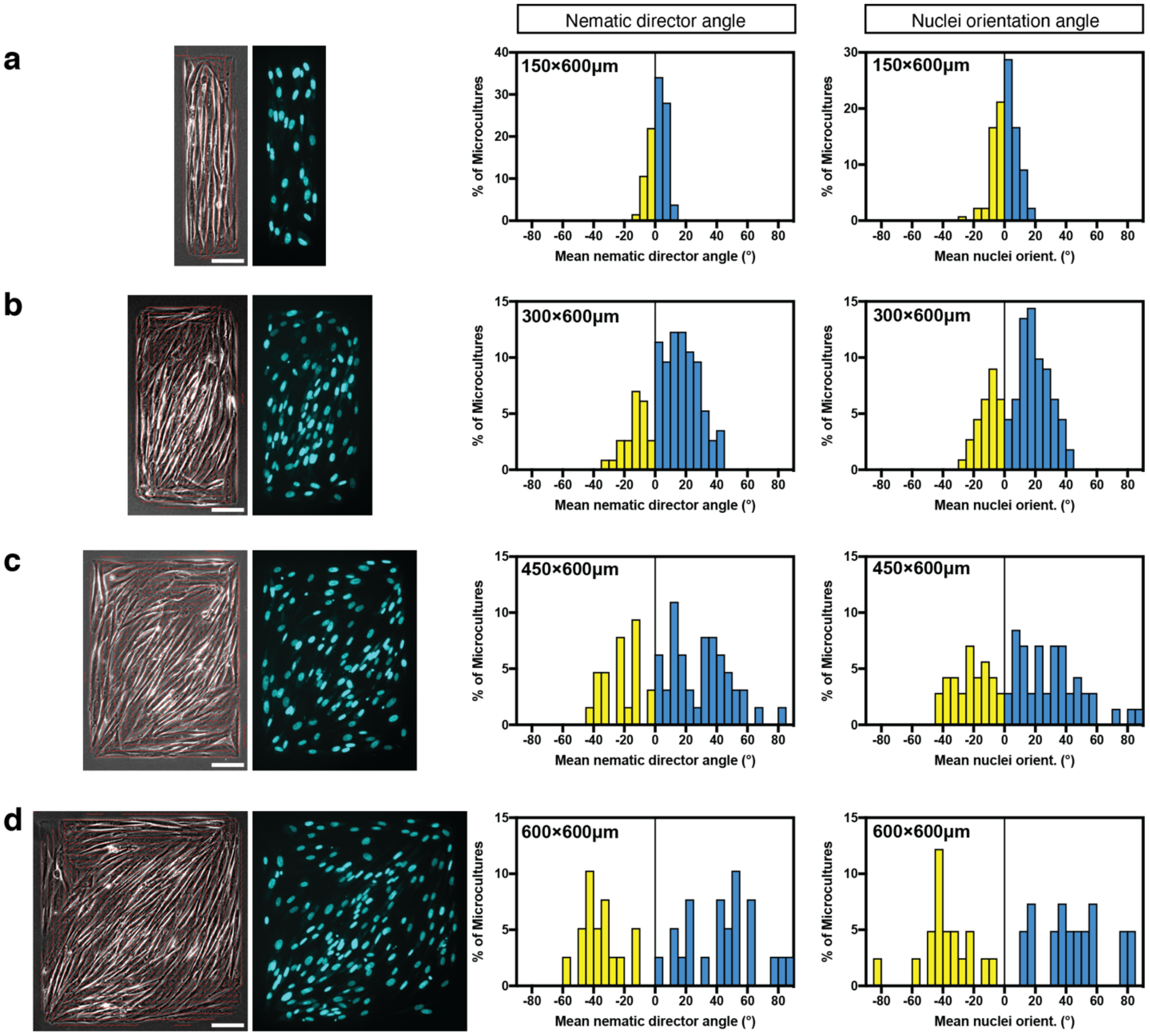

Supplementary Figure 3. Relationship between aspect ratio of rectangular micropattern and left-right asymmetric cell alignment in microcultures.

a-d Phase-contrast image overlaid with local nematic directors (red lines) (left) and the corresponding image of cell nuclei stained with Hoechst 33342 (right) of microcultures on 150×600 (**a**), 300×600 (**b**), 450×600 (**c**) and 600×600 (**d**) µm rectangular micropatterns. Histograms showing distributions of the values of mean nematic directors angle and mean nuclei orientation characterising individual microcultures on rectangles under respective conditions. The histograms were built based on average local cell orientation (nematic directors angle) values from a single experiment comprising 132 (**a**), 114 (**b**), 64 (**c**) and 39 (**d**) microcultures, or average nuclei orientation values from 132 (**a**), 111 (**b**), 71 (**c**) and 41 (**d**) microcultures respectively. Negative and positive values are coloured in yellow and cyan respectively. Scale bars, 100 µm (**a-d**).

a

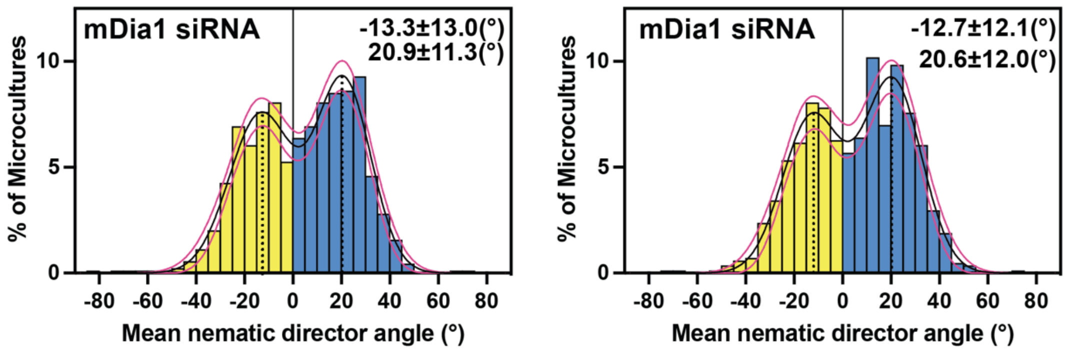

b

Comparison of Fits	
Null hypothesis	Gaussian
Alternative hypothesis	Sum of two Gaussians

				Representation as a sum of 2 gaussian distribution				95% CI (profile likelihood)	
Figure	Parameter	Condition	P value	Mean1	SD1	Mean2	SD2	95% CI (Mean1)	95% CI (Mean2)
2d	Mean nematic director angle	mDia1 siRNA	<0.0001	-13.25	12.96	20.9	11.25	-15.73 to -10.28	18.86 to 22.92
2d	Mean nuclei orient.	mDia1 siRNA	<0.0001	-12.73	12.1	20.64	11.98	-15.38 to -9.318	18.27 to 23.13
Supp Fig. 2g	Mean nematic director angle	FMNL2 siRNA	<0.0001	-9.25	11.66	21.44	12.97	-13.45 to -0.02236	18.09 to 26.37
Supp Fig. 2h	Mean nematic director angle	mDia2 siRNA	<0.0001	-1.437	17.8	28.97	7.549	-5.886 to 5.028	27.16 to 30.54
4c	Mean nematic director angle	20nM LatA	<0.0001	-26.87	10.11	1.255	14.41	-28.46 to -25.28	-11.11 to 7.739
4c	Mean nematic director angle	5nM SwinA	<0.0001	-19.4	15.68	18.96	11.67	-21.47 to -18.03	13.75 to 22.02
Supp Fig. 7e	Mean nuclei orient.	5nM SwinA	<0.0001	-24.69	12.93	7.947	16.21	-27.49 to -22.16	-5.788 to 14.52
7f	Mean nematic director angle	Pfn1 & αActn1 siRNAs	0.0002	-4.384	15.14	27.82	8.323	-10.36 to 5.993	22.82 to 31.88
7g	Mean nematic director angle	CapZβ siRNA	<0.0001	-16.84	11.61	16.16	10.02	-23.81 to -12.16	3.446 to 20.28
Supp Fig. 7e	Mean nuclei orient.	CapZβ siRNA	<0.0001	-19.14	10.23	12.3	11.85	-21.67 to -16.57	6.811 to 16.15
7d	Mean RF tilt angle	LatA + αActn1 siRNA	0.0043	-7.736	12.56	19.38	5.023	-9.934 to -5.357	16.73 to 21.84

Supplementary Figure 4. Fitting of histogram as a sum of two Gaussian distributions.
a The distribution of angles of mean nematic director angle (left) or mean nuclei orientation (right) characterising mDia1 knockdown cells alignment in microcultures shows bimodality. The fit as a sum of two Gaussians distribution is represented by the black line bound by a pair of magenta lines that define the 95% confidence bands. The means for each individual Gaussian distribution is indicated by the black dotted line on the graph and their values (mean±SD) are shown at the top right corner. Negative and positive values are coloured in yellow and cyan respectively. See also Fig. 2d.
b A list summarising other conditions that satisfied the fit as a sum of two Gaussians as defined by a comparison of fits using a null hypothesis (Gaussian fit) versus an alternative hypothesis (Sum of two Gaussians fit) as implemented in GraphPad Prism Software (version 9.4.1). Bimodal distribution is represented by 1 negative (Mean1) and 1 positive (Mean2) means corresponding to opposite chirality signs. See Methods for more details.

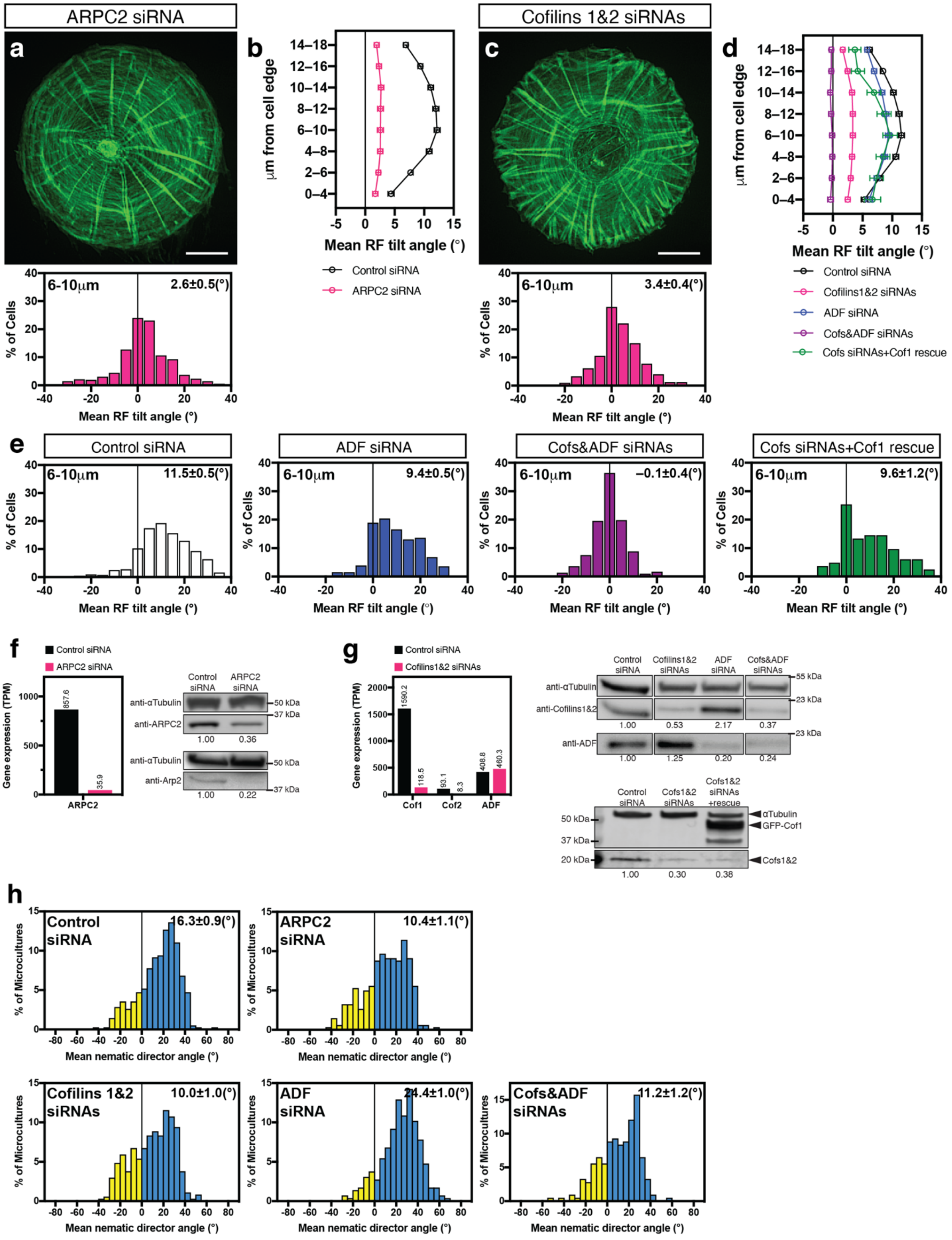

Supplementary Figure 5. Quantification of left-right asymmetry in actin organisation and chiral alignment of cells with knockdown of ARPC2 and actin-depolymerisation factor

(ADF)/cofilin family proteins.
a-d Actin organisation visualised by phalloidin staining in ARPC2 siRNA (**a**) and Cofilins 1 and 2 siRNAs (**c**) transfected cells 6 hours following cell plating on circular pattern. The histograms (**a** and **c**) show the distribution of average radial fibre (RF) tilt in the 6–10 μm annulus in cells under corresponding conditions. The graphs (**b** and **d**) show the average values of RF tilts (mean±SEM) at 6 hours after plating as a function of the distance of annuli from the cell edge for experiments with ARPC2 (**b**) and ADF/Cofilins (**d**) respectively. Scale bars, 10 μm (**a** and **c**).
e The histograms showing the distribution of average RF tilt in the 6–10 μm annulus in cells transfected as indicated at 6 hours after plating. Colour coding in histograms (**c** and **e**) correspond to those indicated in graph (**d**).
f siRNA knockdown of ARPC2 in fibroblasts as verified by RNA-profiling (left) and western blot (right upper). Level of Arp2 is also reduced in ARPC2 siRNA transfected cells as compared to control cells (right lower).
g siRNA knockdown of ADF/Cofilins family proteins as verified by RNA-profiling (left) and western blot (right upper). Rescue of Cofilins 1&2 knockdown cells by co-transfection with GFP-Cofilin 1 full-length plasmid is shown in lane 3 of western blot (right lower). Fold change in protein level relative to loading control (α-Tubulin) and normalised to protein expression in control cells (expression ratio = 1.00) are indicated at the bottom of each blot result in (**f**) and (**g**).
h Quantification of chiral alignment of cells in microcultures at 48 hours after plating under corresponding conditions as characterised by mean nematic directors angle. Mean±SEM values are indicated at the top right corner of each histogram. Negative and positive values are coloured in yellow and cyan respectively.
Sample sizes (n) for (**a**) to (**e**) and (**h**) can be found in Supplementary Table 2. See uncropped blots in Source Data. For statistical analysis, see Supplementary Table 1, lines 167–187.

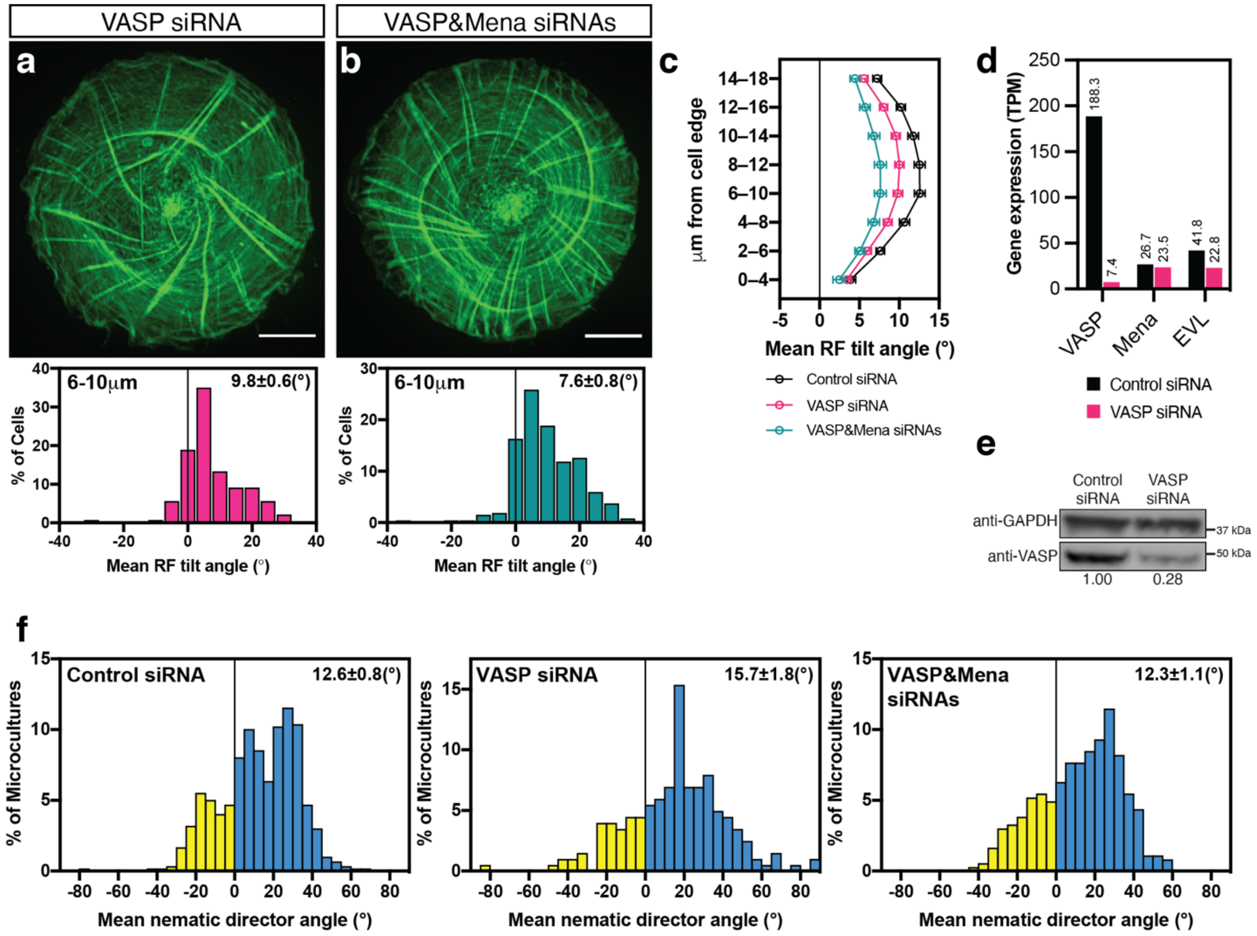

Supplementary Figure 6. Effect of knockdown of actin filament elongator VASP on left-right asymmetry of actin organisation in individual cells and chiral cell alignment in microcultures.

a,b Actin organisation visualised by phalloidin staining in VASP siRNA (**a**) and VASP and Mena siRNAs (**b**) transfected cells 6 hours following cell plating on circular pattern. The histograms show the distribution of average RF tilt in the 6–10 μm annulus in cells at 6 hours after plating under corresponding conditions. Scale bars, 10 μm.

c Average values of RF tilts (mean±SEM) as a function of the distance of annuli from the cell edge. Histograms and mean±SEM values of these average RF tilts (in **a-c**) were obtained from 214 control cells, 271 VASP knockdown cells and 143 VASP and Mena knockdown cells. Colour coding in histograms (**a** and **b**) correspond to those indicated in graph (**c**).

d Transcriptome profiling of gene expression levels (transcripts per million; TPM) of the Ena/VASP family proteins by RNA-sequencing in control- and VASP- siRNA transfected human fibroblasts.

e Western blot showing VASP level in cells treated with scrambled (control) or anti-VASP siRNA; GAPDH was used as a loading control. Fold change in VASP protein level relative to

loading control and normalised to protein expression level in control cells (expression ratio = 1.00) are indicated at the bottom of the blot. See uncropped blots in Source Data.

f Histograms showing distributions of the values of mean nematic directors angle characterising individual microcultures on rectangles under corresponding conditions. The histograms and mean± SEM values were built based on average local cell orientation (nematic directors) values from 598 control, 202 VASP knockdown and 366 VASP and Mena knockdown microcultures. Negative and positive values are coloured in yellow and cyan respectively.

For statistical analysis, see Supplementary Table 1, lines 188–196.

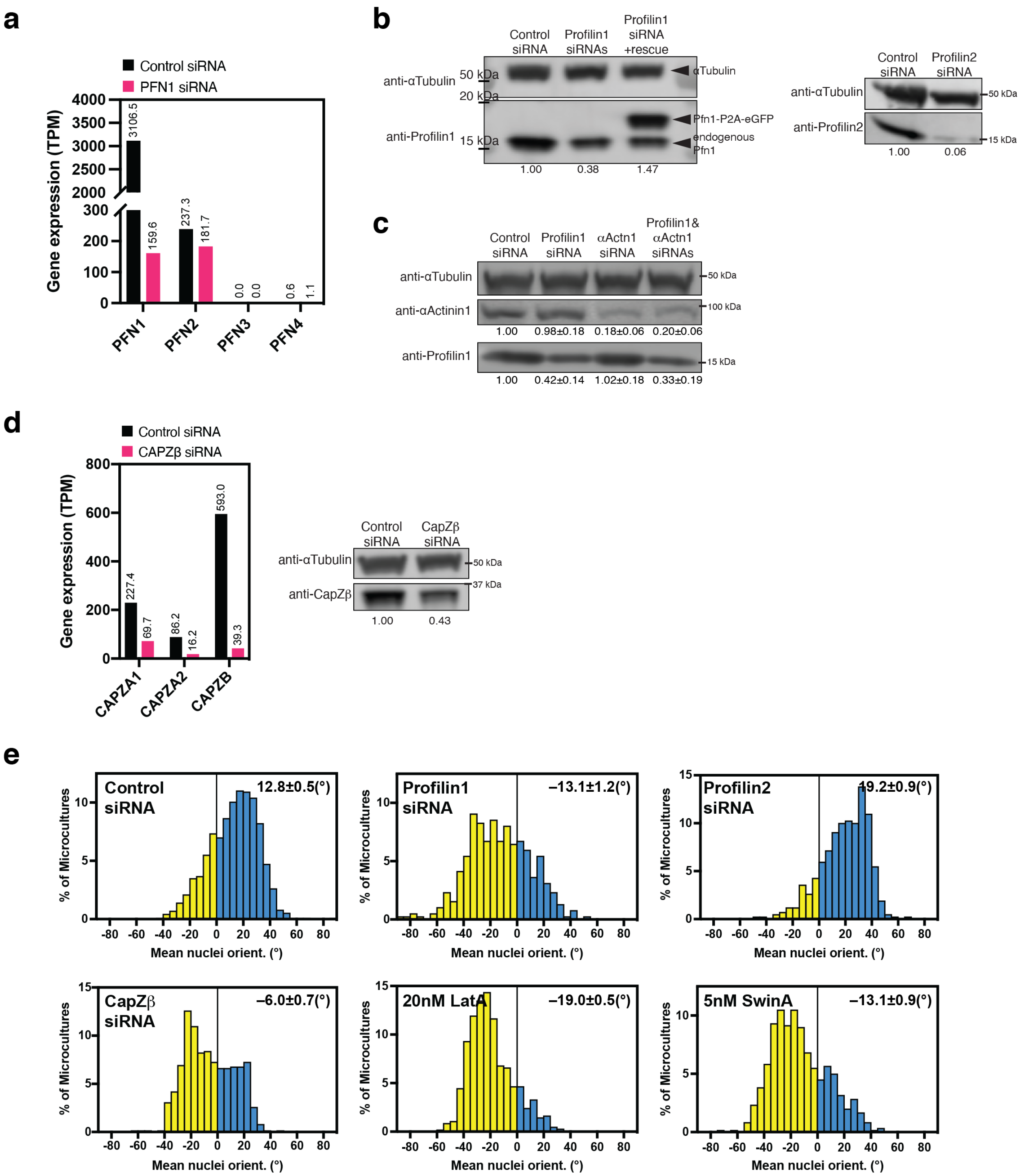

Supplementary Figure 7. Knockdown of profilin 1 and CapZβ protein and their effect on nuclei orientation of cells in microcultures.

a Transcriptome profiling of gene expression levels (transcripts per million; TPM) of Profilin (PFN) 1-4 isoforms identified by RNA-sequencing in control siRNA and profilin 1 siRNA transfected cells.

b Western blots showing profilin 1 (left) and profilin 2 (right) levels in cells treated with scrambled (control), profilin 1 siRNA or profilin 2 siRNA; α-tubulin was used as a loading control. Rescue of profilin 1 knockdown cells by co-transfection with Pfn1-P2A-eGFP full-length plasmid is shown in lane 3 of western blot (left).
c Expression level of profilin 1 and α−actinin1 (αActn1) in single and double siRNAs transfected cells were examined (mean±SD; n= 3 experiments).
d Transcriptome profiling of gene expression levels of CapZA1, CapZA2 and CapZB identified by RNA-sequencing in control siRNA and CapZβ siRNA transfected cells. Western blot showing CapZβ levels in cells treated with scrambled (control) or CapZβ siRNA; α-tubulin was used as a loading control. Fold change in protein level relative to loading control (α-Tubulin) and normalised to protein expression in control cells (expression ratio = 1.00) are indicated at the bottom of each blot results in **b**-**d**. See uncropped blots in Source Data.
e Quantification of chiral alignment of cells in microcultures under respective conditions as characterised by mean nuclei orientation. The histograms were built based on average nuclei orientation values from 1144 control, 386 profilin 1 knockdown, 420 profilin 2 knockdown, 620 CapZβ knockdown microcultures and 1031 LatA- and 601 SwinA-treated microcultures. Mean±SEM values are indicated at the top right corner of each histogram. Negative and positive values are coloured in yellow and cyan respectively. See also Fig. 4**c**.
For statistical analysis, see Supplementary Table 1, lines 197–207.

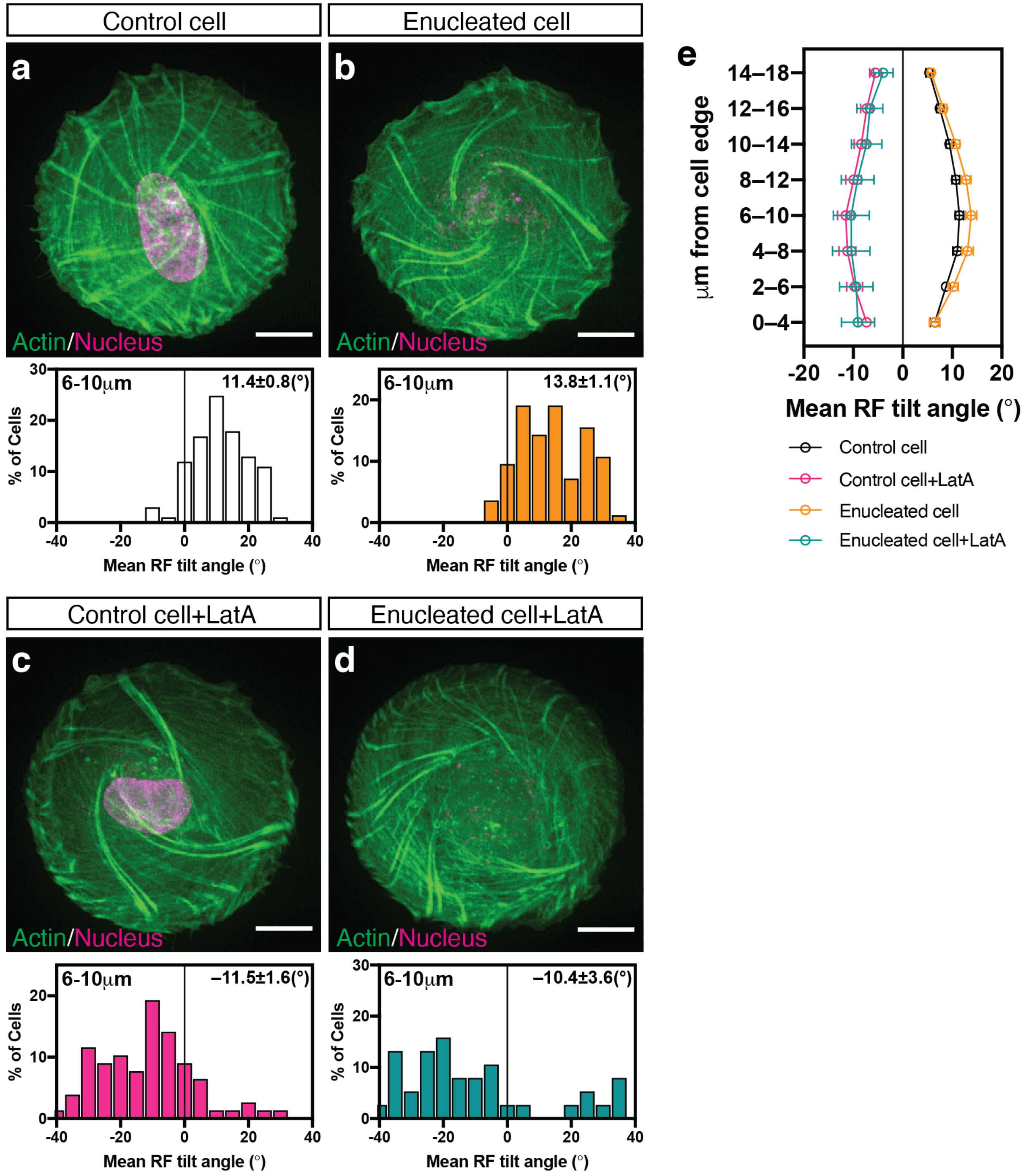

Supplementary Figure 8. Evaluation of chirality in enucleated and latrunculin A-treated enucleated cells.

a-d Actin organisation (green) as visualised by LifeAct-GFP and nuclei as labelled by Hoechst 33342 (pseudo-coloured magenta) in control cell (**a**), enucleated cell (**b**), control cell treated with 20nM Latrunculin A (LatA) (**c**) and enucleated cell treated with 20nM LatA (**d**). The histograms

(**a-d**) show the distribution of average RF tilt in the 6–10 μm annulus in cells under corresponding conditions. Scale bars, 10 μm (**a-d**).

e Average values of RF tilts (mean±SEM) as a function of the distance of annuli from the cell edge. Histograms and mean±SEM values of these average RF tilts (in **a-e**) were calculated from 101 control cells, 84 enucleated cells, 78 LatA-treated control cells and 38 LatA-treated enucleated cells. Colour coding in histograms (**a-d**) correspond to those indicated in graph (**e**). See also Supplementary Movie 6. For statistical analysis, see Supplementary Table 1, lines 208–215.

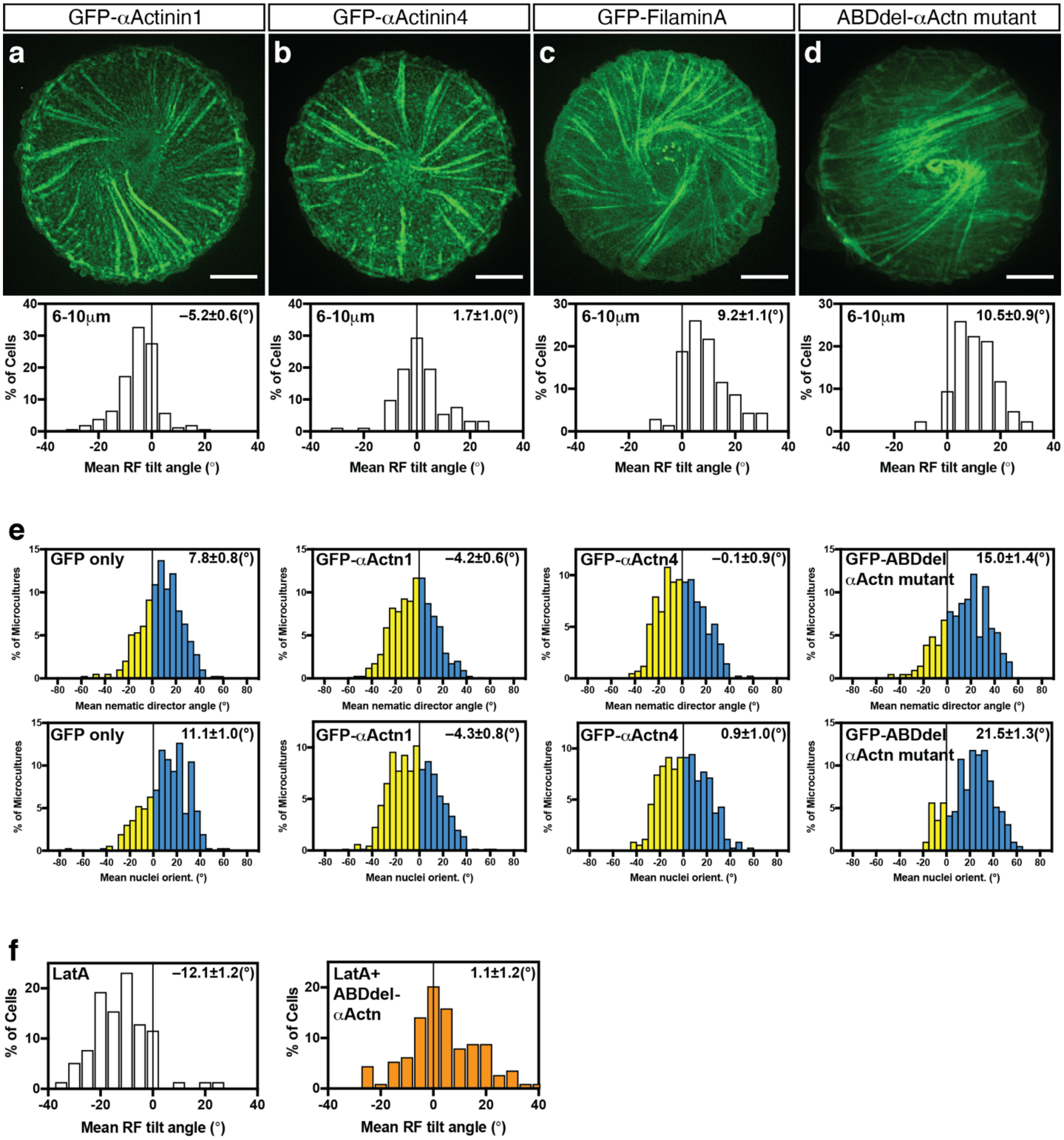

Supplementary Figure 9. Effects of overexpression of α−actinin1 and other crosslinking proteins on radial fibre (RF) tilt and cell alignment in microcultures.

a Image of GFP-α−actinin1 expressing cell showing clockwise actin organisation (as shown in Fig. 6**a**).

b Image of GFP-α−actinin4 expressing cell showing radial actin organisation.

c Image of GFP-Filamin A expressing cell showing anti-clockwise actin organisation.

d Anti-clockwise actin organisation in GFP-ABDdel-α–actinin mutant expressing cell visualised by mRuby-LifeAct (pseudo-coloured green) (as shown in Fig. 6**e**). The histograms (**a-d**) show the distribution of average RF tilt in the 6–10 μm annulus in cells under corresponding conditions. See also Fig. 6**b** and 6**f**. Scale bars, 10 μm (**a-d**).
e Quantification of chiral alignment of cells transfected as indicated in microcultures as characterised by mean nematic directors angle (upper row) or mean nuclei orientation (lower row). The histograms were built based on average local cell orientation (nematic directors) values from 394 GFP-only transfected, 745 GFP-α–actinin1 transfected, 417 GFP-α–actinin4 transfected and 206 GFP-ABDdel-α–actinin mutant transfected microcultures respectively, or average nuclei orientation values from 364 GFP-only transfected cells, 659 GFP-α–actinin1 transfected, 350 GFP-α–actinin4 transfected and 195 GFP-ABDdel-α–actinin mutant transfected microcultures respectively. Histograms depicting the distribution of mean nematic directors angle of GFP-only, GFP-α–actinin1 and GFP-ABDdel-α–actinin mutant expressing cells are also shown in Fig. 6**d** and 6**h**. Negative and positive values are coloured in yellow and cyan respectively.
f Effect of inhibition of α–actinin1 crosslinking function by GFP-ABDdel-α–actinin mutant on reversal of RF tilt by latrunculin A (LatA) treatment. Histograms showing the distribution of average RF tilt in the 6–10 μm annulus in 20nM LatA-treated cells (left) (n= 78 cells) versus GFP-ABDdel-α–actinin mutant expressing cells treated with 20nM LatA (right) (n= 114 cells). Mean±SEM values are indicated at the top right corner of each histogram.
For statistical analysis, see Supplementary Table 1, lines 56–81 and 216–218.

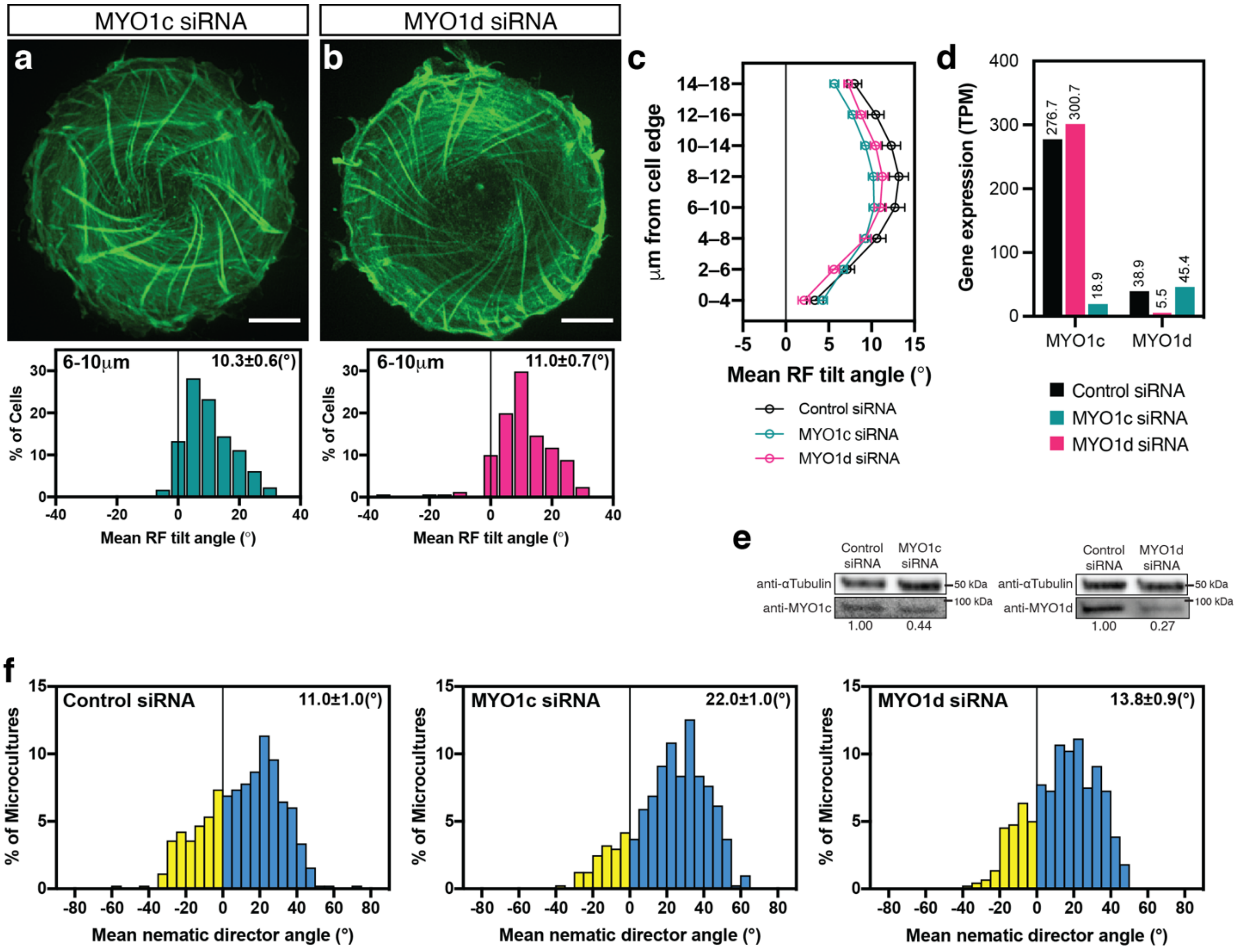

Supplementary Figure 10. Left-right asymmetry of actin organisation and chiral cell alignment in microcultures of myosin 1c and 1d knockdown cells.

a,b Actin organisation visualised by phalloidin labelling in myosin 1c (MYO1c) siRNA (**a**) and myosin 1d (MYO1d) siRNA (**b**) transfected cells 6 hours following cell plating on circular pattern. The histograms show the distribution of average RF tilt in the 6–10 μm annulus in cells under corresponding conditions. Scale bars, 10 μm.

c Average values of RF tilts (mean±SEM) as a function of the distance of annuli from the cell edge. Colour coding in histograms (**a** and **b**) corresponds to those indicated in graph (**c**).

d Transcriptome profiling of gene expression levels (transcripts per million; TPM) of MYO1c and MYO1d identified by RNA-sequencing in control-, MYO1c- and MYO1d- siRNA transfected human fibroblasts.

e Western blots showing MYO1c (left) or MYO1d (right) level in cells treated with scrambled (control), anti-MYO1c or anti-MYO1d siRNA; α-tubulin was used as loading controls. Fold change in protein level relative to loading control (α-Tubulin) and normalised to protein expression in control cells (expression ratio = 1.00) are indicated at the bottom of each blot.

f Quantification of chiral alignment of cells in microcultures as characterised by mean nematic directors. Negative and positive values are coloured in yellow and cyan respectively.
Mean±SEM values are indicated at the top right corner of each histogram.
Sample sizes (n) for (**a-c**) and (**f**) can be found in Supplementary Table 2. For statistical analysis, see Supplementary Table 1, lines 219–227.

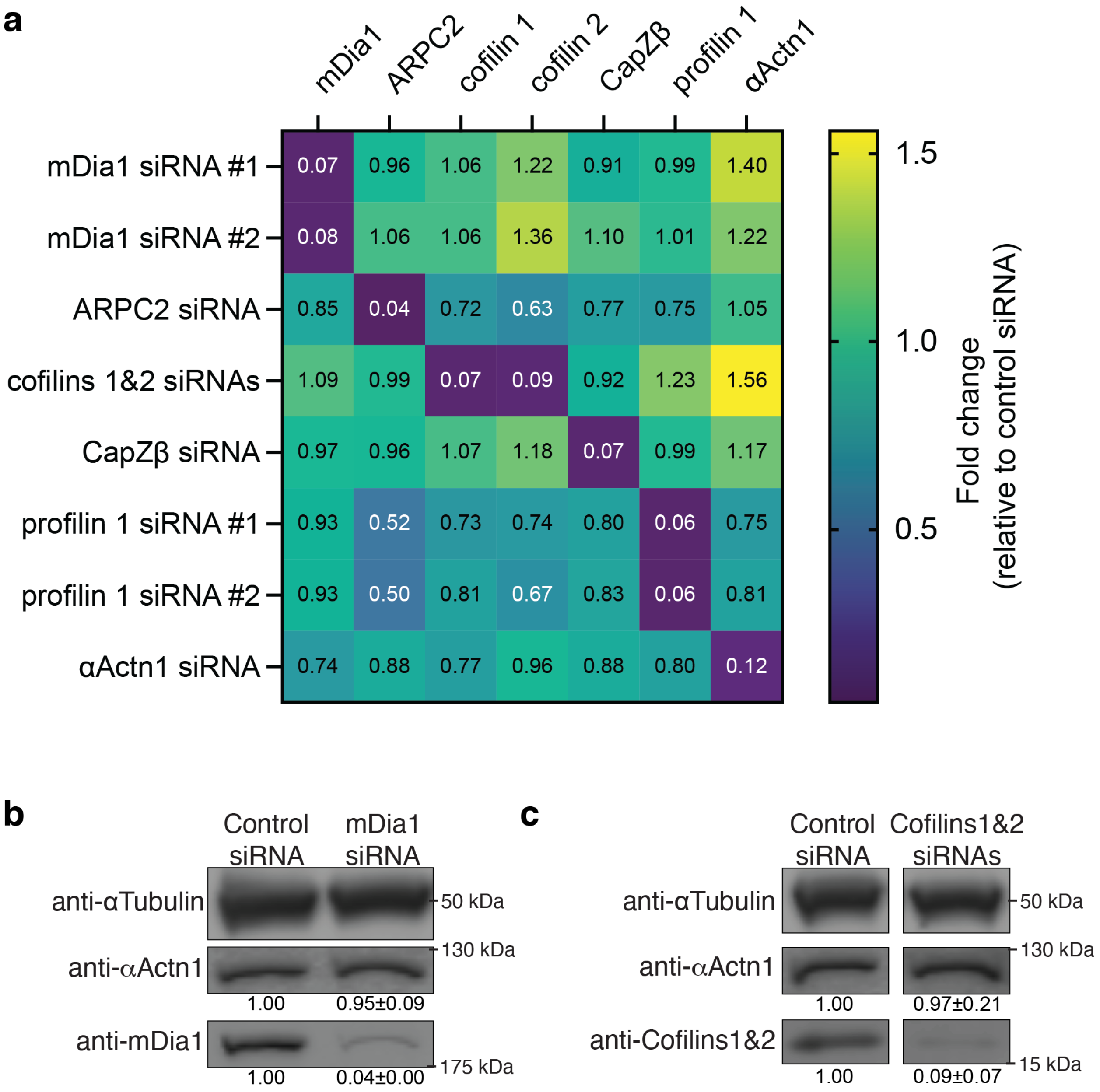

Supplementary Figure 11. Transcriptional profile of major proteins associated with chiral actin organisation.

a Fold changes in gene expression levels (indicated by colour coding and numbers) of major proteins under specific knockdown conditions as assessed by RNA-sequencing. #1 and #2 shows the results of two individual experiments with the same siRNA.

b,c Western blots showing mDia1 (**b**), cofilins 1&2 (**c**) and α–actinin-1 (**b,c**) protein level in cells treated with scrambled (control) (**b,c**), anti-mDia1 (**b**) or anti-cofilins 1&2 (**c**) siRNAs; α-tubulin was used as loading control. Quantification of fold change relative to control was indicated as mean±SD values for 3 experiments.

See measured transcripts per million (TPM) values and uncropped blots in Source Data.

a

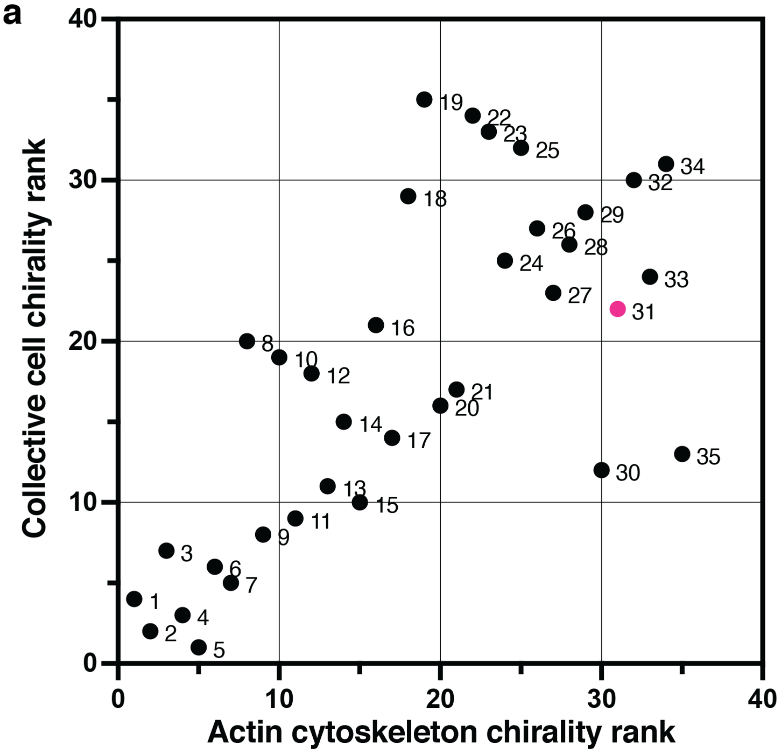

Rank (*x*-axis)	Condition (s)	Rank (*y*-axis)
1	5nM SwinA	4
2	20nM LatA	2
3	GFP-αActn1	7
4	Profilin1 siRNA	3
5	αActn1 siRNA + 20nM LatA	1
6	CAPZβ siRNA	6
7	Profilin1&2 siRNAs	5
8	COFs & ADF siRNAs	20
9	GFP-αActn4	8
10	ARPC2 siRNA	19
11	mDia1 siRNA	8
12	Cofilins1&2 siRNAs	18
13	PFNs & αActn1 siRNAs	11
14	FMNL2 siRNA	15
15	FHOD3 siRNA	10
16	VASP & Mena siRNAs	21
17	DAAM1 siRNA	14
18	FMN2 siRNA	29
19	ADF siRNA	35
20	mDia3 siRNA	16
21	mDia2 siRNA	17
22	INF2 siRNA	34
23	MYO1c siRNA	33
24	GFP-ABDdel αActn mutant	25
25	CAPZβ & αActn1 siRNAs	32
26	VASP siRNA	27
27	MYO1d siRNA	23
28	FHOD1 siRNA	26
29	Daam2 siRNA	28
30	Pfn1 & αActn1 siRNAs	12
31	Control	22
32	αActn1&4 siRNAs	30
33	Pfn2 siRNA	24
34	αActn1 siRNA	31
35	αActn4 siRNA	13

b

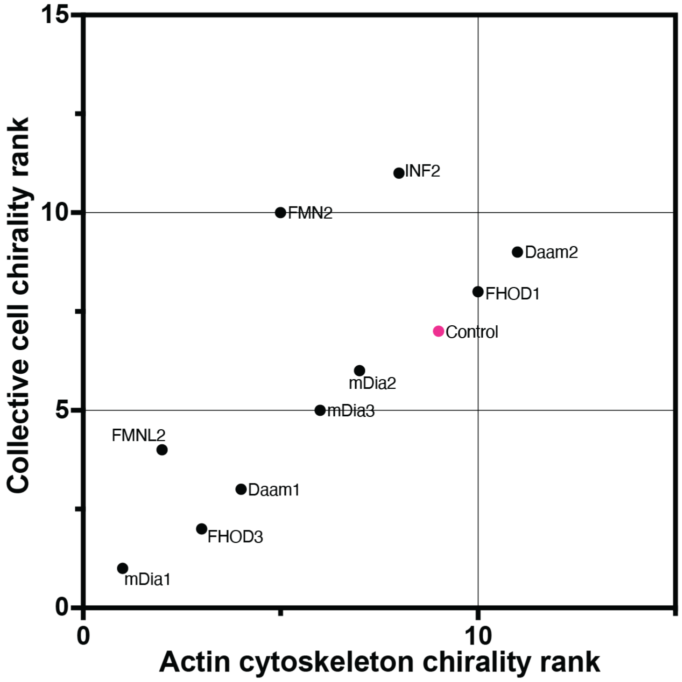

Rank (*x*-axis)	Condition (s)	Rank (*y*-axis)
1	mDia1 siRNA	1
2	FMNL2 siRNA	4
3	FHOD3 siRNA	2
4	DAAM1 siRNA	3
5	FMN2 siRNA	10
6	mDia3 siRNA	5
7	mDia2 siRNA	6
8	INF2 siRNA	11
9	Control	7
10	FHOD1 siRNA	8
11	DAAM2 siRNA	9

Supplementary Figure 12. The rank correlation between actin cytoskeleton chirality in individual cells and collective cell chirality in microcultures.

a Each numbered dot represents average data from pooled experiments under respective conditions indicated in the list on the right. All dots are ranked according to actin cytoskeleton

chirality value and collective chirality value. Actin cytoskeleton chirality is defined as the mean radial fibre tilt angle at the 6–10 μm annulus. Collective chirality is defined as the mean nematic director angle for rectangular microcultures. Dots are indexed in ascending actin cytoskeleton chirality rank (*x*-axis). Position of control cell (31) is shown in magenta. Numbers of cells and microcultures analysed and the corresponding mean values can be found in Supplementary Table 2. See also Fig. 8. Spearman's rank correlation coefficient, r = 0.7081, ****p<0.0001.
b The rank correlation between actin cytoskeleton chirality in individual cells and collective cell chirality in microculture for the cells with knockdowns of formin family members. The ranking of the chirality values was performed in the same way as in (**a**) and indicated in the list on the right. Position of control cell is shown in magenta. Spearman's rank correlation coefficient, r = 0.7545, **p=0.0098.

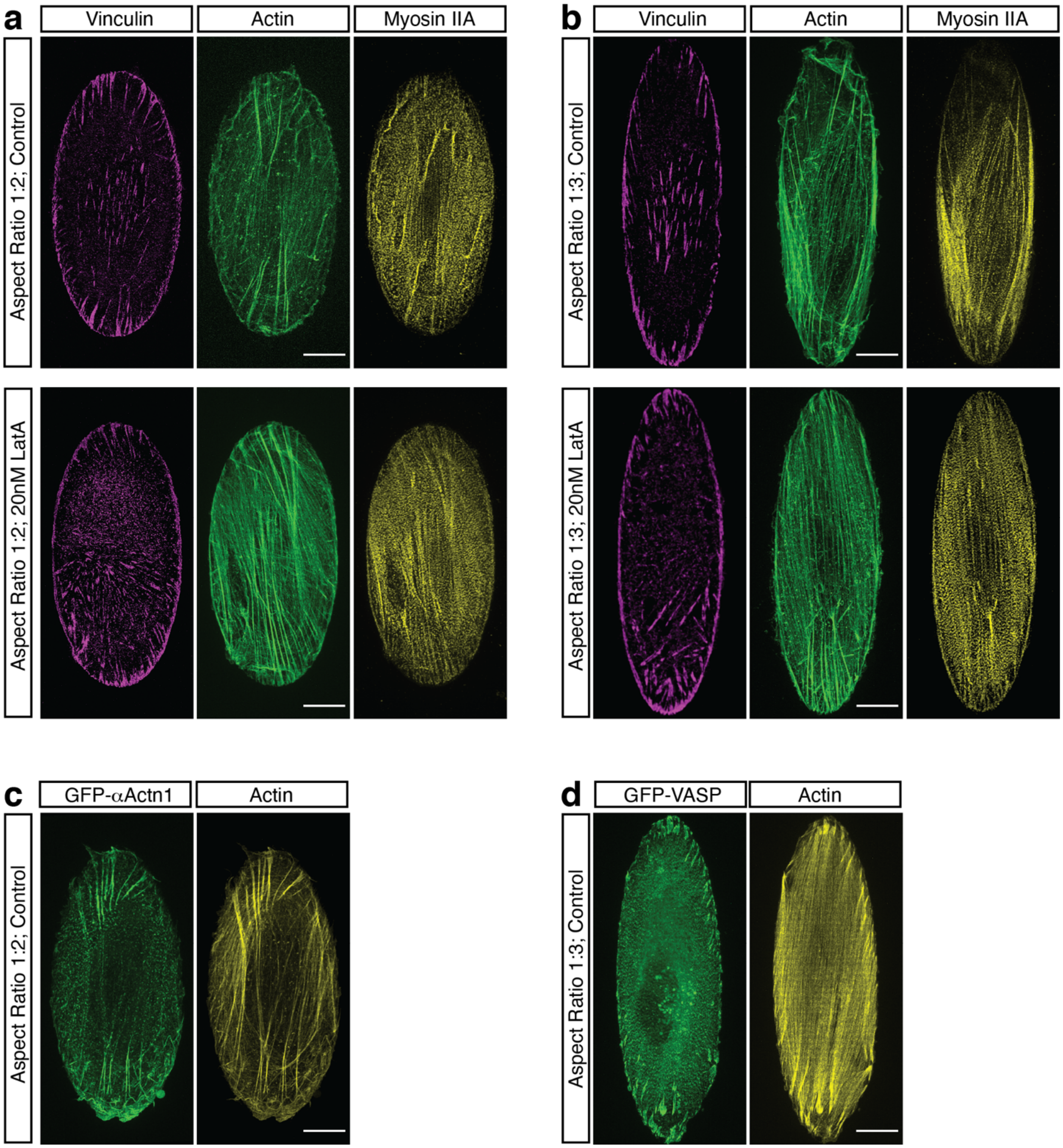

Supplementary Figure 13. The system of chiral stress fibres in cells on an elliptical pattern.
a,b Focal adhesions were visualised by anti-vinculin antibody staining, actin fibres by phalloidin staining and myosin IIA by anti-myosin-IIA antibody staining in cells fixed at 6 hours after spreading on an elliptical micropattern with different aspect ratios. Cells were either untreated (control) or treated with 20nM latrunculin A (LatA).
c,d Actin fibres were visualised by phalloidin staining, α–actinin1 (αActn1) by transfection with GFP-αActn1 (**c**) and VASP by transfection with GFP-VASP (**d**) in control cells on elliptical

micropatterns. GFP-αActn1 (**c**) strongly localised to radial fibres while GFP-VASP (**d**) localised to focal adhesions. Note the right-handed and left-handed tilt of actomyosin stress fibres relative to the long axis of the ellipses in control and latrunculin-treated cells respectively.
Scale bars, 10 μm (**a-d**). See also Fig. 9.

Supplementary Table 1. Statistical analysis of data.

Line	Figure	Statistical Test	Parameters	Comparison	P-value	P-value summary
1	1i vs. 1j	Kruskal-Wallis	Mean RF tilt angle	Control siRNA vs. mDia1 siRNA	<0.0001	****
2	1j vs. 1k			mDia1 siRNA vs. mDia1 siRNA+rescue	<0.0001	****
3	1i vs. 1k			Control siRNA vs. mDia1 siRNA+rescue	>0.9999	ns
4	1i	Wilcoxon test	Mean RF tilt angle	Median of Control siRNA vs. zero	<0.0001	****
5	1j			Median of mDia1 siRNA vs. zero	<0.0001	****
6	1k			Median of mDia1 siRNA+rescue vs. zero	<0.0001	****
7	2d	Kruskal-Wallis	Mean nematic director angle	Control siRNA vs. mDia1 siRNA	<0.0001	****
8	2d		Mean nuclei orient.	Control siRNA vs. mDia1 siRNA	<0.0001	****
9	2d		Mean nematic director angle vs. Mean nuclei orient.	Control siRNA vs. Control siRNA	>0.9999	ns
10	2d			mDia1 siRNA vs. mDia1 siRNA	>0.9999	ns
11	2d	Wilcoxon test	Mean nematic director angle	Median of Control siRNA vs. zero	<0.0001	****
12	2d		Mean nematic director angle	Median of mDia1 siRNA vs. zero	<0.0001	****
13	2d		Mean nuclei orient.	Median of Control siRNA vs. zero	<0.0001	****
14	2d		Mean nuclei orient.	Median of mDia1 siRNA vs. zero	<0.0001	****
15	3a vs. 3b	Kruskal-Wallis	Mean RF tilt angle	Control siRNA vs. Profilin1 siRNA	<0.0001	****
16	3b vs. 3c			Pfn1 siRNA vs. Profilin1 siRNA+rescue	<0.0001	****
17	3a vs. 3c			Control siRNA vs. Profilin1 siRNA+rescue	>0.9999	ns
18	3a vs. 3d			Control siRNA vs. Profilin2 siRNA	<0.0001	****
19	3a	Wilcoxon test	Mean RF tilt angle	Median of Control siRNA vs. zero	<0.0001	****
20	3b			Median of Profilin1 siRNA vs. zero	<0.0001	****
21	3c			Median of Profilin1 siRNA+rescue vs. zero	<0.0001	****
22	3d			Median of Profilin2 siRNA vs. zero	<0.0001	****
23	3f vs. 3e	Mann-Whitney	Mean RF tilt angle (8 - 12μm)	Control siRNA #2 vs. CapZβ siRNA	<0.0001	****
24	3e	Wilcoxon test	Mean RF tilt angle (8 - 12μm)	Median of CapZβ siRNA vs. zero	<0.0001	****
25	3h vs. 3g	Kruskal-Wallis	Mean RF tilt angle	Untreated Control vs. 20nM LatA	<0.0001	****
26	3h			Untreated Control vs. 5nM SwinA	<0.0001	****
27	3h	Wilcoxon test	Mean RF tilt angle	Median of Untreated Control vs. zero	<0.0001	****
28	3g			Median of 20nM LatA vs. zero	<0.0001	****
29	3h			Median of 5nM SwinA vs. zero	<0.0001	****
30	4c	Kruskal-Wallis	Mean nematic director angle	Control siRNA vs. 20nM LatA	<0.0001	****
31	4c			Control siRNA vs. Profilin1 siRNA	<0.0001	****
32	4c			Control siRNA vs. Profilin2 siRNA	0.7157	ns
33	4c			Control siRNA vs. CapZβ siRNA	<0.0001	****
34	4c			Control siRNA vs. 5nM SwinA	<0.0001	****
35	4c	Wilcoxon test	Mean nematic director angle	Median of Control siRNA vs. zero	<0.0001	****
36	4c			Median of 20nM LatA vs. zero	<0.0001	****
37	4c			Median of Profilin1 siRNA vs. zero	<0.0001	****
38	4c			Median of Profilin2 siRNA vs. zero	<0.0001	****
39	4c			Median of CapZβ siRNA vs. zero	<0.0001	****
40	4c			Median of 5nM SwinA vs. zero	<0.0001	****
41	5a vs. 5b	Kruskal-Wallis	Mean RF tilt angle	Control siRNA vs. Profilin1 siRNA	<0.0001	****
42	5a vs. 5c			Control siRNA vs. Control siRNA+LatA	<0.0001	****
43	5a vs. 5d			Control siRNA vs. mDia1 siRNA	<0.0001	****
44	5a vs. 5e			Control siRNA vs. mDia1&Profilin1 siRNAs	<0.0001	****
45	5a vs. 5f			Control siRNA vs. mDia1 siRNA+LatA	<0.0001	****
46	5d vs. 5e			mDia1 siRNA vs. mDia1&Profilin1 siRNAs	<0.0001	****
47	5d vs. 5f			mDia1 siRNA vs. mDia1 siRNA+LatA	<0.0001	****
48	5e vs. 5b			mDia1&Profilin1 siRNAs vs. Profilin1 siRNA	>0.9999	ns
49	5f vs. 5c			mDia1 siRNA+LatA vs. Control siRNA+LatA	0.3911	ns
50	5a	Wilcoxon test	Mean RF tilt angle	Median of Control siRNA vs. zero	<0.0001	****
51	5b			Median of Profilin1 siRNA vs. zero	<0.0001	****
52	5c			Median of siControl siRNA+LatA vs. zero	<0.0001	****
53	5d			Median of mDia1 siRNA vs. zero	<0.0001	****
54	5e			Median of mDia1&Profilin1 siRNAs vs. zero	<0.0001	****
55	5f			Median of mDia1 siRNA+LatA vs. zero	<0.0001	****
56	6b/Supp Fig. 9a	Kruskal-Wallis	Mean RF tilt angle	Control vs. GFP-αActn1	<0.0001	****
57	6b/Supp Fig. 9b			Control vs. GFP-αActn4	<0.0001	****
58	6b/Supp Fig. 9c			Control vs. GFP-FlnA	>0.9999	ns
59	6b	Wilcoxon test	Mean RF tilt angle	Median of Control vs. zero	<0.0001	****
60	6b/Supp Fig. 9a			Median of GFP-αActn1 vs. zero	<0.0001	****
61	6b/Supp Fig. 9b			Median of GFP-αActn4 vs. zero	>0.9999	ns
62	6b/Supp Fig. 9c			Median of GFP-FlnA vs. zero	<0.0001	****
63	6d/Supp Fig. 9e	Kruskal-Wallis	Mean nematic director angle	GFP only control vs. GFP-αActn1	<0.0001	****
64	Supp Fig. 9e			GFP only control vs. GFP-αActn4	<0.0001	****
65	Supp Fig. 9e			GFP only control vs. GFP-ABDdel αActn mutant	0.0016	**
66	Supp Fig. 9e		Mean nuclei orient.	GFP only control vs. GFP-αActn1	<0.0001	****
67	Supp Fig. 9e			GFP only control vs. GFP-αActn4	<0.0001	****
68	Supp Fig. 9e			GFP only control vs. GFP-ABDdel αActn mutant	<0.0001	****
69	6d/Supp Fig. 9e	Wilcoxon test	Mean nematic director angle	Median of GFP only control vs. zero	<0.0001	****
70	6d/Supp Fig. 9e			Median of GFP-αActn1 vs. zero	<0.0001	****
71	Supp Fig. 9e			Median of GFP-αActn4 vs. zero	0.7214	ns
72	6h/Supp Fig. 9e			Median of GFP-ABDdel-αActn1 mutant vs. zero	<0.0001	****
73	Supp Fig. 9e		Mean nuclei orient.	Median of GFP control vs. zero	<0.0001	****
74	Supp Fig. 9e			Median of GFP-αActn1 vs. zero	<0.0001	****
75	Supp Fig. 9e			Median of GFP-αActn4 vs. zero	0.5323	ns
76	Supp Fig. 9e			Median of GFP-ABDdel-αActn1 mutant vs. zero	<0.0001	****
77	6f	Kruskal-Wallis	Mean RF tilt angle	Control siRNA vs. αActn1 siRNA	0.1425	ns
78	6f/Supp Fig. 9d			Control siRNA vs. ABDdel-αActn mutant	0.0003	***
79	6f	Wilcoxon test	Mean RF tilt angle	Median of Control siRNA vs. zero	<0.0001	****
80	6f			Median of αActn1 siRNA vs. zero	<0.0001	****
81	6f/Supp Fig. 9d			Median of ABDdel-αActn1 mutant vs. zero	<0.0001	****
82	7a	Kruskal-Wallis	Mean RF tilt angle	Control siRNA vs. αActn1 siRNA	>0.9999	ns
83	7b			Pfn1 siRNA vs. Pfn1&αActn1 siRNAs	<0.0001	****
84	7a vs. 7b			Control siRNA vs. Pfn1 siRNA	<0.0001	****
85	7a vs. 7b			Control siRNA vs. Pfn1&αActn1 siRNAs	>0.9999	ns
86	7d			LatA vs. LatA+αActn1 siRNAs	<0.0001	****
87	7a vs.7d			Control siRNA vs. LatA	<0.0001	****
88	7a vs.7d			Control siRNA vs. LatA+αActn1 siRNAs	<0.0001	****
89	7c		Mean RF tilt angle (8 - 12μm)	CapZβ siRNA vs. CapZβ&αActn1 siRNAs	<0.0001	****
90	7a vs. 7c			Control siRNA vs. CapZβ siRNA	<0.0001	****
91	7a vs. 7c			Control siRNA vs. CapZβ&αActn1 siRNAs	0.0005	***
92	7a	Wilcoxon test	Mean RF tilt angle	Median of Control siRNA vs. zero	<0.0001	****
93	7a			Median of αActn1 siRNA vs. zero	<0.0001	****
94	7b			Median of Pfn 1 siRNA vs. zero	<0.0001	****
95	7b			Median of Pfn1&αActn1 siRNAs vs. zero	<0.0001	****
96	7d			Median of LatA vs. zero	<0.0001	****
97	7d			Median of LatA+αActn1 siRNA vs. zero	0.1225	ns
98	7c		Mean RF tilt angle (8 - 12μm)	Median of CapZβ siRNA vs. zero	0.0105	*
99	7c			Median of CapZβ&αActn1 siRNAs vs. zero	<0.0001	****
100	7e	Kruskal-Wallis	Mean nematic director angle	Control siRNA vs. αActn1 siRNA	<0.0001	****
101	7f			Pfn1 siRNA vs. Pfn1&αActn1 siRNAs	<0.0001	****
102	7e vs. 7f			Control siRNA vs. Pfn1 siRNA	<0.0001	****
103	7e vs. 7f			Control siRNA vs. Pfn1&αActn1 siRNAs	>0.9999	ns
104	7g			CapZβ siRNA vs. CapZβ&αActn1 siRNAs	<0.0001	****
105	7e vs. 7g			Control siRNA vs. CapZβ siRNA	<0.0001	****

106	7e vs. 7g			Control siRNA vs. CapZβ&αActn1 siRNAs	<0.0001	****
107	7h			LatA vs. LatA+αActn1 siRNAs	>0.9999	ns
108	7e vs. 7h			Control siRNA vs. LatA	<0.0001	****
109	7e vs. 7h			Control siRNA vs. LatA+αActn1 siRNA	<0.0001	****
110	7e			Median of Control siRNA vs. zero	<0.0001	****
111	7e			Median of αActn1 siRNA vs. zero	<0.0001	****
112	7f			Median of Pfn1 siRNA vs. zero	<0.0001	****
113	7f	Wilcoxon test	Mean nematic director angle	Median of Pfn1&αActn1 siRNAs vs. zero	<0.0001	****
114	7g			Median of CapZβ siRNA vs. zero	0.0028	**
115	7g			Median of CapZβ&αActn1 siRNAs vs. zero	<0.0001	****
116	7h			Median of LatA vs. zero	<0.0001	****
117	7h			Median of LatA+αActn1 siRNA vs. zero	<0.0001	****
118		Mann-Whitney		Control vs. 20nM LatA (Aspect Ratio 1:2)	<0.0001	****
119	9c	Wilcoxon test	Stress Fibre Tilt	Median of Control vs. zero (Aspect Ratio 1:2)	<0.0001	****
120				Median of 20nM LatA vs. zero (Aspect Ratio 1:2)	<0.0001	****
121		Mann-Whitney		Control vs. 20nM LatA (Aspect Ratio 1:3)	<0.0001	****
122	9f	Wilcoxon test	Stress Fibre Tilt	Median of Control vs. zero (Aspect Ratio 1:3)	<0.0001	****
123				Median of 20nM LatA vs. zero (Aspect Ratio 1:3)	<0.0001	****
124				Control siRNA vs. mDia1 siRNA	<0.0001	****
125	Supp Fig. 2b			Control siRNA vs. FMNL2 siRNA	<0.0001	****
126				Control siRNA vs. FHOD3 siRNA	<0.0001	****
127				Control siRNA vs. Daam1 siRNA	<0.0001	****
128				Control siRNA vs. FMN2 siRNA	0.1131	ns
129				Control siRNA vs. mDia3 siRNA	>0.9999	ns
130	Supp Fig. 2c	Kruskal-Wallis	Mean RF tilt angle	Control siRNA vs. mDia2 siRNA	>0.9999	ns
131				Control siRNA vs. INF2 siRNA	>0.9999	ns
132				Control siRNA vs. FHOD1 siRNA	>0.9999	ns
133				Control siRNA vs. Daam2 siRNA	>0.9999	ns
134				mDia1 siRNA vs. FMNL2 siRNA	<0.0001	****
135	Supp Fig. 2b			mDia1 siRNA vs. FHOD3 siRNA	<0.0001	****
136				mDia1 siRNA vs. Daam1 siRNA	<0.0001	****
137				Median of Control siRNA vs. zero	<0.0001	****
138				Median of mDia1 siRNA vs. zero	<0.0001	****
139	Supp Fig. 2b			Median of FMNL2 siRNA vs. zero	<0.0001	****
140				Median of FHOD3 siRNA vs. zero	<0.0001	****
141				Median of Daam1 siRNA vs. zero	<0.0001	****
142		Wilcoxon test	Mean RF tilt angle	Median of FMN2 siRNA vs. zero	<0.0001	****
143				Median of mDia3 siRNA vs. zero	<0.0001	****
144	Supp Fig. 2c			Median of mDia2 siRNA vs. zero	<0.0001	****
145				Median of INF2 siRNA vs. zero	<0.0001	****
146				Median of FHOD1 siRNA vs. zero	<0.0001	****
147				Median of Daam2 siRNA vs. zero	<0.0001	****
148				Control siRNA vs. FMNL2 siRNA	0.0027	**
149	Supp Fig. 2g			Control siRNA vs. FHOD3 siRNA	<0.0001	****
150				Control siRNA vs. Daam1 siRNA	0.0425	*
151				Control siRNA vs. FMN2 siRNA	>0.9999	ns
152		Kruskal-Wallis	Mean nematic director angle	Control siRNA vs. mDia3 siRNA	>0.9999	ns
153	Supp Fig. 2h			Control siRNA vs. mDia2 siRNA	0.054	ns
154				Control siRNA vs. INF2 siRNA	<0.0001	****
155				Control siRNA vs. FHOD1 siRNA	>0.9999	ns
156				Control siRNA vs. Daam2 siRNA	>0.9999	ns
157				Median of Control siRNA vs. zero	<0.0001	****
158	Supp Fig. 2g			Median of FMNL2 siRNA vs. zero	<0.0001	****
159				Median of FHOD3 siRNA vs. zero	<0.0001	****
160				Median of Daam1 siRNA vs. zero	<0.0001	****
161		Wilcoxon test	Mean nematic director angle	Median of FMN2 siRNA vs. zero	<0.0001	****
162				Median of mDia3 siRNA vs. zero	0.0044	**
163	Supp Fig. 2h			Median of mDia2 siRNA vs. zero	<0.0001	****
164				Median of INF2 siRNA vs. zero	<0.0001	****
165				Median of FHOD1 siRNA vs. zero	<0.0001	****
166				Median of Daam2 siRNA vs. zero	<0.0001	****
167	Supp Fig. 5a	Mann-Whitney	Mean RF tilt angle	Control siRNA vs. ARPC2 siRNA	<0.0001	****
168		Wilcoxon test		Median of ARPC2 siRNA vs. zero	<0.0001	****
169	Supp Fig. 5, e vs. c			Control siRNA vs. Cofilins 1&2 siRNAs	<0.0001	****
170	Supp Fig. 5, c vs. e			Cofilins1&2 siRNAs vs. Cofs siRNAs+Cof1 rescue	0.0001	***
171		Kruskal-Wallis	Mean RF tilt angle	Control siRNA vs. Cofs siRNAs+Cof1 rescue	0.2418	ns
172	Supp Fig. 5e			Control siRNA vs. ADF siRNA	0.0386	*
173				Control siRNA vs. Cofs&ADF siRNAs	<0.0001	****
174	Supp Fig. 5c			Median of Cofilins1&2 siRNAs vs. zero	<0.0001	****
175				Median of Control siRNA vs. zero	<0.0001	****
176	Supp Fig. 5e	Wilcoxon test	Mean RF tilt angle	Median of ADF siRNA vs. zero	<0.0001	****
177				Median of Cofs&ADF siRNAs vs. zero	0.9554	ns
178				Median of Cofs siRNAs+Cof1 rescue vs. zero	0.0001	***
179				Control siRNA vs. ARPC2 siRNA	0.0004	***
180	Supp Fig. 5h	Kruskal-Wallis	Mean nematic director angle	Control siRNA vs. Cofilins1&2 siRNAs	<0.0001	****
181				Control siRNA vs. ADF siRNA	<0.0001	****
182				Control siRNA vs. Cofs&ADF siRNAs	0.0046	**
183				Median of Control siRNA vs. zero	<0.0001	****
184				Median of ARPC2 siRNA vs. zero	<0.0001	****
185	Supp Fig. 5h	Wilcoxon test	Mean nematic director angle	Median of Cofilins1&2 siRNAs vs. zero	<0.0001	****
186				Median of ADF siRNA vs. zero	<0.0001	****
187				Median of Cofs&ADF siRNAs vs. zero	<0.0001	****
188	Supp Fig. 6, c vs. a	Kruskal-Wallis	Mean RF tilt angle	Control siRNA vs. VASP siRNA	0.0167	*
189	Supp Fig. 6, c vs. b			Control siRNA vs. VASP&Mena siRNAs	<0.0001	****
190	Supp Fig. 6a	Wilcoxon test	Mean RF tilt angle	Median of VASP siRNA vs. zero	<0.0001	****
191	Supp Fig. 6b			Median of VASP&Mena siRNAs vs. zero	<0.0001	****
192	Supp Fig. 6f	Kruskal-Wallis	Mean nematic director angle	Control siRNA vs. VASP siRNA	0.3084	ns
193				Control siRNA vs. VASP&Mena siRNAs	>0.9999	ns
194				Median of Control siRNA vs. zero	<0.0001	****
195	Supp Fig. 6f	Wilcoxon test	Mean nematic director angle	Median of VASP siRNA vs. zero	<0.0001	****
196				Median of VASP&Mena siRNAs vs. zero	<0.0001	****
197				Control siRNA vs. Profilin1 siRNA	<0.0001	****
198				Control siRNA vs. Profilin2 siRNA	0.0002	***
199	Supp Fig. 7e	Kruskal-Wallis	Mean nuclei orient.	Control siRNA vs. CapZβ siRNA	<0.0001	****
200				Control siRNA vs. 20nM LatA	<0.0001	****
201				Control siRNA vs. 5nM SwinA	<0.0001	****
202				Median of Control siRNA vs. zero	<0.0001	****
203				Median of Profilin 1 siRNA vs. zero	<0.0001	****
204	Supp Fig. 7e	Wilcoxon test	Mean nuclei orient.	Median of Profilin 2 siRNA vs. zero	<0.0001	****
205				Median of CapZβ siRNA vs. zero	<0.0001	****
206				Median of 20nM LatA vs. zero	<0.0001	****
207				Median of 5nM SwinA vs. zero	<0.0001	****
208	Supp Fig. 8, a vs. b			Control cell vs. Enucleated cell	>0.9999	ns
209	Supp Fig. 8, a vs. c	Kruskal-Wallis	Mean RF tilt angle	Control cell vs. Control cell+LatA	<0.0001	****
210	Supp Fig. 8, c vs. d			Control cell+LatA vs. Enucleated cell+LatA	>0.9999	ns
211	Supp Fig. 8, b vs. d			Enucleated cell vs. Enucleated cell+LatA	<0.0001	****
212	Supp Fig. 8a			Median of Control cell	<0.0001	****
213	Supp Fig. 8b	Wilcoxon test	Mean RF tilt angle	Median of Enucleated cell	<0.0001	****
214	Supp Fig. 8c			Median of Control cell+LatA	<0.0001	****
215	Supp Fig. 8d			Median of Enucleated cell+LatA	0.0109	*

216	Supp Fig. 9f	Mann-Whitney	Mean RF tilt angle	LatA vs. LatA+ABDdel αActn mutant	<0.0001	****
217		Wilcoxon test		Median of 20nM LatA vs. zero	<0.0001	****
218				Median of LatA+ABDdel αActn mutant vs. zero	0.616	ns
219	Supp Fig. 10, c vs. a	Kruskal-Wallis	Mean RF tilt angle	Control siRNA vs. MYO1c siRNA	>0.9999	ns
220	Supp Fig. 10, c vs. b			Control siRNA vs. MYO1d siRNA	0.0783	ns
221	Supp Fig. 10a	Wilcoxon test	Mean RF tilt angle	Median of MYO1c siRNA vs. zero	<0.0001	****
222	Supp Fig. 10b			Median of MYO1d siRNA vs. zero	<0.0001	****
223	Supp Fig. 10f	Kruskal-Wallis	Mean nematic director angle	Control siRNA vs. MYO1c siRNA	<0.0001	****
224				Control siRNA vs. MYO1d siRNA	0.2975	ns
225	Supp Fig. 10f	Wilcoxon test	Mean nematic director angle	Median of Control siRNA vs. zero	<0.0001	****
226				Median of MYO1c siRNA vs. zero	<0.0001	****
227				Median of MYO1d siRNA vs. zero	<0.0001	****
228	1j vs. 5d	Kruskal-Wallis	Mean RF tilt angle	mDia1 siRNA vs. mDia1 siRNA	>0.9999	ns
229	1i vs. 5a			Control siRNA vs. Control siRNA	0.0001	***
230	3b vs. 5b	Kruskal-Wallis	Mean RF tilt angle	Profilin1 siRNA vs. Profilin1 siRNA	>0.9999	ns
231	3b vs. 7b			Profilin1 siRNA vs. Pfn1 siRNA	0.4581	ns
232	5b vs. 7b			Profilin1 siRNA vs. Pfn1 siRNA	0.0748	ns
233	3a vs. 5a			Control siRNA vs. Control siRNA	0.0289	*
234	3a vs. 7a			Control siRNA vs. Control siRNA	0.5144	ns
235	5a vs. 7a			Control siRNA vs. Control siRNA	>0.9999	ns
236	3e vs. 7c	Kruskal-Wallis	Mean RF tilt angle (8 - 12μm)	CapZβ siRNA vs. CapZβ siRNA	>0.9999	ns
237	3f vs. 7a			Control siRNA #2 vs. Control siRNA	>0.9999	ns
238	3g vs. 5c	Kruskal-Wallis	Mean RF tilt angle	20nM LatA vs. Control siRNA+LatA	>0.9999	ns
239	3g vs. 7d			20nM LatA vs. LatA	>0.9999	ns
240	3g vs. Supp Fig. 8c			20nM LatA vs. Control cell+LatA	>0.9999	ns
241	5c vs. 7d			Control siRNA+LatA vs. LatA	>0.9999	ns
242	5c vs. Supp Fig. 8c			Control siRNA+LatA vs. Control cell+LatA	>0.9999	ns
243	7d vs. Supp Fig. 8c			LatA vs. Control cell+LatA	>0.9999	ns
244	3h vs. 5a			Untreated Control vs. Control siRNA	<0.0001	****
245	3h vs. 7a			Untreated Control vs. Control siRNA	0.0085	**
246	3h vs. Supp Fig. 8a			Untreated Control vs. Control cell	>0.9999	ns
247	5a vs. 7a			Control siRNA vs. Control siRNA	>0.9999	ns
248	5a vs. Supp Fig. 8a			Control siRNA vs. Control cell	0.1523	ns
249	7a vs. Supp Fig. 8a			Control siRNA vs. Control cell	>0.9999	ns
250	4c vs. 7e	Kruskal-Wallis	Mean nematic director angle	Control siRNA vs. Control siRNA	>0.9999	ns
251	4c vs. 7f			Profilin1 siRNA vs. Pfn1 siRNA	0.1574	ns
252	4c vs. 7g			CapZβ siRNA vs. CapZβ siRNA	0.0422	*
253	4c vs. 7h			20nM LatA vs. LatA	<0.0001	****

All statistical tests were implemented using Graphpad Prism software (version 9.4.1).
Statistical significance is defined as P<0.05 for all tests.
Two-tailed Mann-Whitney test was used.
Kruskal-Wallis test was implemented with Dunn's multiple comparisons test and report multiplicity adjusted P value for each comparison.
Wilcoxon signed-rank test was implemented against a hypothetical value of zero.

See Methods for details of computation of mean radial fibre (RF) tilt angle of individual cells and mean nematic director angle and mean nuclei orient. of cell collectives in microcultures.
Parameter: Mean radial fibre (RF) tilt angle refers to measurements in the 6–10 μm annuli in cells (unless otherwise stated).

Supplementary Table 2. Mean radial fibre (RF) tilt angles at the 6-10 μm annulus and corresponding mean nematic director angles for rectangular microcultures for each type of treatment. Ranks and sample sizes (n) are included. See also Fig. 8 and Supplementary Fig. 12.

Rank of RF tilt angle	Condition(s)	Mean RF tilt angle (°)	Mean nematic director angle (°)	Rank of nematic director angle	n= (cells)	n= (micro-cultures)
1	5nM SwinA	-11.80±0.78	-13.43±0.70	4	153	1108
2	20nM LatA	-10.24±0.48	-22.47±0.54	2	490	1416
3	GFP-αActn1	-5.18±0.60	-4.15±0.65	7	156	745
4	Profilin1 siRNA	-4.59±0.51	-17.50±0.77	3	431	715
5	αActn1 + 20nM LatA	-2.38±1.55	-26.24±1.20	1	94	237
6	CAPZβ siRNA	-2.21±0.52	-7.56±0.72	6	409	661
7	Profilins1&2 siRNAs	-0.37±1.08	-8.59±1.38	5	76	207
8	COFs & ADF siRNAs	-0.11±0.41	11.16±1.19	20	308	216
9	GFP-αActn4	1.68±0.97	-0.07±0.88	8	92	417
10	ARPC2 siRNA	2.59±0.53	10.38±1.05	19	434	342
11	mDia1 siRNA	2.78±0.36	4.02±0.71	9	515	894
12	Cofilins1&2 siRNA	3.37±0.43	10.00±0.99	18	426	373
13	PFNs & αActn1 siRNAs	4.45±0.76	6.39±1.34	11	295	227
14	FMNL2 siRNA	6.66±0.64	8.87±0.95	15	227	454
15	FHOD3 siRNA	7.08±0.64	5.99±0.87	10	262	407
16	VASP & Mena siRNAs	7.63±0.76	12.26±1.10	21	143	366
17	DAAM1 siRNA	7.78±0.70	8.21±1.44	14	268	165
18	FMN2 siRNA	7.97±0.92	16.02±1.49	29	115	181
19	ADF siRNA	9.38±0.54	24.36±1.01	35	339	295
20	mDia3 siRNA	9.58±0.99	9.05±2.93	16	98	32

21	mDia2 siRNA	9.93±0.60	9.41±1.04	17	283	419
22	INF2 siRNA	9.95±0.53	22.56±0.74	34	356	495
23	MYO1c siRNA	10.29±0.58	21.99±0.98	33	181	406
24	GFP-ABDdel αActn mutant	10.51±0.87	14.99±1.37	25	85	206
25	CAPZβ & αActn1 siRNAs	10.69±0.73	18.73±0.78	32	219	360
26	VASP siRNA	10.84±0.46	15.70±1.78	27	395	202
27	MYO1d siRNA	11.01±0.69	13.78±0.86	23	171	440
28	FHOD1 siRNA	11.45±0.91	15.03±1.26	26	147	157
29	DAAM2 siRNA	11.66±1.10	15.92±1.25	28	100	215
30	Pfn1 & αActn1 siRNAs	11.84±1.33	7.90±1.67	12	42	135
31	Control	11.85±0.25	12.56±0.28	22	1559	4432
32	αActn1&4 siRNAs	15.47±1.22	16.19±1.07	30	105	182
33	Profilin2 siRNA	17.16±0.63	14.98±0.76	24	182	519
34	αActn1 siRNA	17.35±0.62	17.61±0.78	31	264	430
35	αActn4 siRNA	18.00±0.70	8.20±1.47	13	116	135

Supplementary Table 3. List of siRNAs used.

siRNA	Company, Product name or Target Sequence(s)	Catalogue No.
Control	Dharmacon, ON-TARGETplus Non-targeting control	D-001810-01
Alpha-Actinin1	Dharmacon, ON-TARGETplus SMARTpool, Human ACTN1 siRNA	L-011195-00
Alpha-Actinin4	Dharmacon, ON-TARGETplus SMARTpool, Human ACTN4 siRNA	L-011988-00
ADF	Dharmacon, ON-TARGETplus, Human DSTN siRNA	J-012303-05 & J-012303-06
ARPC2	Dharmacon, ON-TARGETplus SMARTpool, Human ARPC2 siRNA	L-012081-00
CapZβ	Dharmacon, ON-TARGETplus SMARTpool, Human CAPZB siRNA	L-011990-00
Cofilin 1	Santa Cruz Biotechnology Inc, Cofilin 1 siRNA (h)	sc-35078
Cofilin 2	Santa Cruz Biotechnology Inc, Cofilin 2 siRNA (h)	sc-37027
DAAM1	Santa Cruz Biotechnology Inc, DAAM1 siRNA (h)	sc-62190
DAAM2	Santa Cruz Biotechnology Inc, DAAM2 siRNA (h)	sc-62192
FHOD1	Santa Cruz Biotechnology Inc, FHOD1 siRNA (h)	sc-60635
FHOD3	Dharmacon, ON-TARGETplus SMARTpool, Human FHOD3 siRNA	L-023411-01
FMNL2	Dharmacon, ON-TARGETplus, Human FMNL2 siRNA	J-031993-09 & J-031993-10
FMN2	Santa Cruz Biotechnology Inc, Formin 2 siRNA (h)	sc-43765
INF2	Santa Cruz Biotechnology Inc, INF2 siRNAs (h)	sc-92159
mDia1	Dharmacon, ON-TARGETplus, Human DIAPH1 siRNA	J-010347-06
mDia2	Dharmacon, ON-TARGETplus SMARTpool, Human DIAPH3 siRNA	L-018997-00
mDia3	Dharmacon, ON-TARGETplus, Human DIAPH2 siRNA	J-012029-05 & J-012029-06
Mena	Santa Cruz Biotechnology Inc, Mena siRNA (h)	sc-43496
MYO1c	Santa Cruz Biotechnology Inc, Myosin 1c siRNA (h)	sc-44604
MYO1d	Santa Cruz Biotechnology Inc, Myosin 1d siRNA (h)	sc-44608
Profilin 1	5’-GCAAAGACCGGUCAAGUUU-3’ and 5’-CACGGUGGUUUGAUCAACA-3’	
Profilin 2	5’-GUAGAGCAUUGGUUAUAGU-3’ and 5’-CCAGGGACAUUCCAUCAUU-3’	
VASP	Santa Cruz Biotechnology Inc, VASP siRNA (h)	sc-29516

Supplementary Table 4. List of antibodies used.

Target	Company	Catalogue No.	Dilution
α-Tubulin	Sigma-Aldrich	T6199	1:5000
α-Actinin1	US Biological	A0761-02F	1:1000
ADF	Abcam	ab186754	1:1000
ARP2	Santa Cruz Biotechnology Inc	sc-166103 (E-12)	1:1000
ARPC2	Santa Cruz Biotechnology Inc	sc-515754 (F-5)	1:1000
CAPZβ	Abcam	ab175212	1:1000
Cofilins1&2	Santa Cruz Biotechnology Inc	sc-376476 (E-8)	1:1000
DAAM1	Abcam	ab56951	1:1000
DAAM2	Santa Cruz Biotechnology Inc	sc-515129 (E-1)	1:1000
FHOD1	ECM Biosciences	FM3521	1:1000
FMN2	Santa Cruz Biotechnology Inc	sc-376787 (C-3)	1:1000
GAPDH	Santa Cruz Biotechnology Inc	sc-47724 (0411)	1:5000
INF2	Proteintech	20466-1-AP	1:1000
mDia1	BD Biosciences	610849	1:1000
mDia3	ECM Biosciences	DP4511	1:1000
MYO1c	Santa Cruz Biotechnology Inc	sc-136544 (13)	1:1000
MYO1d	Santa Cruz Biotechnology Inc	sc-515292 (H-1)	1:1000
Myosin IIA	Sigma Aldrich	M8064	1:800
Profilin 1	Santa Cruz Biotechnology Inc	sc-137235 (B-10)	1:1000
Profilin 2	Santa Cruz Biotechnology Inc	sc-100955 (4K-6)	1:1000
VASP	Santa Cruz Biotechnology Inc	sc-46668 (A-11)	1:1000
Vinculin	Sigma Aldrich	V9131	1:400
IRDye® 680RD Goat anti-Rabbit IgG	LI-COR, Inc.	926-68071	1: 5000
IRDye® 800CW Goat anti-Mouse IgG	LI-COR, Inc.	926-32210	1:15,000
goat anti-rabbit IgG-HRP	Santa Cruz Biotechnology Inc	sc-2004	1:10,000
goat anti-mouse IgG-HRP	Santa Cruz Biotechnology Inc	sc-2005	1:10,000
Donkey anti-Mouse IgG (H+L) Highly Cross-Adsorbed Secondary Antibody, Alexa Fluor™ 488	Invitrogen	A-21202	1:500
Donkey anti-Rabbit IgG (H+L) Highly Cross-Adsorbed Secondary Antibody, Alexa Fluor™ 647	Invitrogen	A31573	1:500
