# Supplementary material for: Actin polymerisation and crosslinking drive left-right asymmetry in single cell and cell collectives
Source: Nat Commun. 2023 Feb 11;14:776. doi: 10.1038/s41467-023-35918-1 (PMC9922260; doi:10.1038/s41467-023-35918-1)
Supplement: Supplementary file 3 — Description for Additional Supplementary Files [file 41467_2023_35918_MOESM3_ESM.pdf]

Description of additional supplementary files

Movies Captions

Supplementary Movie 1. Anti-clockwise (dextral) chiral actin swirling in control HFF cell confined to circular fibronectin pattern. LifeAct labelling of actin (yellow) and Hoechst 33342 labelling of the nucleus (magenta) are shown. Images were recorded at 20-minute intervals over a period of 14 hours. Display rate is 7 frames/sec.

Supplementary Movie 2. Left-right asymmetric alignment of control HFF cells plated on rectangular fibronectin pattern (300×600 μm). Phase-contrast microscopy. Images were recorded at 2 hours intervals over a period of 56 hours. Display rate is 3 frames/sec.

Supplementary Movie 3. Clockwise (sinistral) chiral actin swirling in profilin 1 siRNA knockdown HFF cell confined to circular fibronectin pattern. LifeAct labelling of actin (yellow) is shown. Images were recorded at 30-minute intervals over a period of 14 hours. Display rate is 7 frames/sec and corresponds to the cell shown in Fig. 3b.

Supplementary Movie 4. Reversal of swirling direction upon addition of 20nM of latrunculin A (LatA). Time of addition of drug is indicated. LifeAct labelling of actin (yellow) is shown. Images were recorded at 2-minute intervals over a period of ~ 2 hours. Display rate is 7 frames/sec.

Supplementary Movie 5. Reversal of swirling direction upon latrunculin A washout. Time of removal of drug is indicated. LifeAct labelling of actin (yellow) is shown. Images were recorded at 3-minute intervals over a period of ~ 4 hours. Display rate is 7 frames/sec.

Supplementary Movie 6. Anti-clockwise actin swirling in enucleated HFF cell (left) and clockwise actin swirling in enucleated HFF cell in the presence of 20nM latrunculin A (right). LifeAct labelling of actin (green) are shown. Images were recorded at 10-minute

intervals over a period of 9.5 hours. Display rate is 7 frames/sec and corresponds to the cells shown in Supplementary Fig. 8, b and d.

Supplementary Movie 7. A typical example of actin cytoskeleton and focal adhesion dynamics as visualised by mRuby-LifeAct (pseudo-colored yellow, left) and GFP-VASP (pseudo-colored magenta, right) respectively in cell on an elliptic (aspect ratio 1:2) fibronectin pattern. Imaging commenced ~ 3 h post-plating and image sequence shown began when cell had adopted fully the elliptic pattern area. Images were recorded at 3-minute intervals over a period of 17 hours. Display rate is 7 frames/sec and corresponds to the time-lapse series in Fig. 9g.

Supplementary Movie 8. Reversal of stress fibre tilt upon addition of 20nM of latrunculin A (LatA). Cell was monitored for an hour at 5-minute interval prior to the addition of latrunculin A. Time of addition of drug was indicated and filming resumed 8 minutes after the introduction of latrunculin A at 8-minute intervals over a period of ~12 hours. LifeAct labelling of actin (green) is shown. Display rate is 10 frames/sec and corresponds to the time-lapse series in Fig. 9, i and j.
