# Supplementary material for: Actin polymerisation and crosslinking drive left-right asymmetry in single cell and cell collectives
Source: Nat Commun. 2023 Feb 11;14:776. doi: 10.1038/s41467-023-35918-1 (PMC9922260; doi:10.1038/s41467-023-35918-1)
Supplement: Supplementary file 12 — Reporting Summary [file 41467_2023_35918_MOESM12_ESM.pdf]

nature portfolio

Corresponding author(s): Yee Han Tee and Alexander D. Bershadsky

Last updated by author(s): Dec 15, 2022

Reporting Summary

Nature Portfolio wishes to improve the reproducibility of the work that we publish. This form provides structure for consistency and transparency in reporting. For further information on Nature Portfolio policies, see our Editorial Policies and the Editorial Policy Checklist.

Statistics

For all statistical analyses, confirm that the following items are present in the figure legend, table legend, main text, or Methods section.

n/a	Confirmed	
[ ]	[x]	The exact sample size (*n*) for each experimental group/condition, given as a discrete number and unit of measurement
[ ]	[x]	A statement on whether measurements were taken from distinct samples or whether the same sample was measured repeatedly
[ ]	[x]	The statistical test(s) used AND whether they are one- or two-sided<br>*Only common tests should be described solely by name; describe more complex techniques in the Methods section.*
[x]	[ ]	A description of all covariates tested
[ ]	[x]	A description of any assumptions or corrections, such as tests of normality and adjustment for multiple comparisons
[ ]	[x]	A full description of the statistical parameters including central tendency (e.g. means) or other basic estimates (e.g. regression coefficient) AND variation (e.g. standard deviation) or associated estimates of uncertainty (e.g. confidence intervals)
[ ]	[x]	For null hypothesis testing, the test statistic (e.g. *F*, *t*, *r*) with confidence intervals, effect sizes, degrees of freedom and *P* value noted<br>*Give P values as exact values whenever suitable.*
[x]	[ ]	For Bayesian analysis, information on the choice of priors and Markov chain Monte Carlo settings
[x]	[ ]	For hierarchical and complex designs, identification of the appropriate level for tests and full reporting of outcomes
[ ]	[x]	Estimates of effect sizes (e.g. Cohen's *d*, Pearson's *r*), indicating how they were calculated

Our web collection on statistics for biologists contains articles on many of the points above.

Software and code

Policy information about availability of computer code

Data collection	MetaMorph (Molecular Devices; versions 7.10.5.476 and 7.10.4.407) or Volocity (Perkin Elmer; version 6.3) commercial softwares were used for image acquisition. The softwares were coupled with the respective microscopes described in the Method section of the manuscript. Image Studio (LiCor; version 1.0.20) or Image Lab Touch (Bio-Rad; version 2.3.0.07) softwares were used to capture western blot results coupled to Odyssey® CLx and GelDoc Go imaging systems respectively.
Data analysis	Image analysis codes are implemented in ImageJ (open source software; versions 2.1.0/1.53c and 2.3.0/1.53f), MatLab (versions R2016b and R2020a) and Python (open source software; version 3.8).<br>(i) Codes for radial fibre segmentation and measurement of their tilt angle can be found at Github - https://github.com/gohweijia/Cell-Chirality-Analysis.<br>(ii) Nematic directors angles were measured using a custom-based script provided by authors of ref. 26.<br>(iii) Custom MATLAB scripts for segmentation of individual rectangular microculture area using phase contrast image and for segmentation and measurement of nuclei orientation angle were described in Methods and available upon request.<br>(iv) Stress fibre tilt angle was quantified using the OrientationJ Measure plugin implemented in ImageJ.<br>GraphPad Prism (version 9.4.1) software was used for data plotting and statistical analyses.

For manuscripts utilizing custom algorithms or software that are central to the research but not yet described in published literature, software must be made available to editors and reviewers. We strongly encourage code deposition in a community repository (e.g. GitHub). See the Nature Portfolio guidelines for submitting code & software for further information.

Data

Policy information about availability of data

All manuscripts must include a data availability statement. This statement should provide the following information, where applicable:

- Accession codes, unique identifiers, or web links for publicly available datasets
- A description of any restrictions on data availability
- For clinical datasets or third party data, please ensure that the statement adheres to our policy

All data generated or analysed during this study are included in this published article (and its Supplementary Information file). Raw datasets corresponding to any of graphs presented in this study are available from the corresponding authors on reasonable request. Gene expression profiles presented as transcript per million (TPM) reads and uncropped western blots are provided as a Source Data file with this paper.

Human research participants

Policy information about studies involving human research participants and Sex and Gender in Research.

Reporting on sex and gender	N/A. No human subjects are involved in this study.
Population characteristics	N/A.
Recruitment	N/A.
Ethics oversight	N/A

Note that full information on the approval of the study protocol must also be provided in the manuscript.

Field-specific reporting

Please select the one below that is the best fit for your research. If you are not sure, read the appropriate sections before making your selection.

☒ Life sciences ☐ Behavioural & social sciences ☐ Ecological, evolutionary & environmental sciences

For a reference copy of the document with all sections, see nature.com/documents/nr-reporting-summary-flat.pdf

Life sciences study design

All studies must disclose on these points even when the disclosure is negative.

Sample size	No statistical method was used to predetermine sample size. Sample size were determined based on whether data is reproducible in independent sets of experiments, typically 30-100 cells or 50-200 microcultures were imaged per experiment and two to three independent experiments are used to determine the reproducibility of data.
Data exclusions	Data exclusions (and rationale behind) are mentioned in the Methods sections, (i) "Measurement of radial fibre tilt angles" and (ii) "Computation of nematic directors and nuclei orientation".<br>For (i), based on visual inspection, we found that actin radial fibre segments with θr more than or equals to 68º were unlikely to be part of radial fibres (but rather of transverse fibres mis-identified by deep-learning analysis) and were thus omitted from the quantification samples. These errors were more often found with the inner annuli (e.g 12-16 and 14-16 µm from the cell edge) and hence our reporting of 6-10 and 8-12 µm from the cell edge is most representative of the samples.<br>For (ii), for each microculture, the centre 200x500 µm region of interest was used for subsequent steps in the measurements of average nuclei orientation and average nematic director orientation. Microcultures that had less than 50 nuclei were removed as these microcultures often had too few cells to cover the entire rectangular micropattern. A mean resultant length cutoff value (see Methods) of 0.35 and 0.5 was selected for nematic director angle and nuclei orientation angle respectively, and only microcultures with a mean resultant length greater than that were quantified.
Replication	The repeated experiments always gave comparable results.
Randomization	In experiments with single cells, randomly-selected cells that filled entirely the circular or elliptical micropatterns were imaged. All rectangular microcultures on each dish were captured by automated acquisition on the microscope.
Blinding	Blinding is not possible. The investigators prepared both control and treated samples at the same time for each independent set of experiments.

Reporting for specific materials, systems and methods

We require information from authors about some types of materials, experimental systems and methods used in many studies. Here, indicate whether each material, system or method listed is relevant to your study. If you are not sure if a list item applies to your research, read the appropriate section before selecting a response.

Materials & experimental systems

n/a	Involved in the study
☐	☒ Antibodies
☐	☒ Eukaryotic cell lines
☒	☐ Palaeontology and archaeology
☒	☐ Animals and other organisms
☒	☐ Clinical data
☒	☐ Dual use research of concern

Methods

n/a	Involved in the study
☒	☐ ChIP-seq
☒	☐ Flow cytometry
☒	☐ MRI-based neuroimaging

Antibodies

Antibodies used

a-Tubulin (Sigma-Aldrich, catalog# T6199, dilution 1:5000); ADF (Abcam, catalog# ab186754, dilution 1:1000); ARP2 (Santa Cruz Biotechnology Inc, catalog# sc-166103 (E-12), dilution 1:1000); ARPC2 (Santa Cruz Biotechnology Inc, catalog# sc-515754 (F-5), dilution 1:1000); CapZb (Abcam, catalog# ab175212, dilution 1:1000); Cofilins1&2 (Santa Cruz Biotechnology Inc, catalog# sc-376476 (E-8), dilution 1:1000); DAAM1 (Abcam, catalog# ab56951, dilution 1:1000); DAAM2 (Santa Cruz Biotechnology Inc, catalog# sc-515129 (E-1), dilution 1:1000); FHOD1 (ECM Biosciences, catalog# FM3521, dilution 1:1000); FMN2 (Santa Cruz Biotechnology Inc, catalog# sc-376787 (C-3), dilution 1:1000); GAPDH (Santa Cruz Biotechnology Inc, catalog# sc-47724 (0411), dilution 1:5000); INF2 (Proteintech, catalog# 20466-1-AP, dilution 1:1000); mDia1 (BD Biosciences, catalog# 610849, dilution 1:1000); mDia3 (ECM Biosciences, catalog# DP4511, dilution 1:1000); MYO1c (Santa Cruz Biotechnology Inc, catalog# sc-136544 (13), dilution 1:1000); MYO1d (Santa Cruz Biotechnology Inc, catalog# sc-515292 (H-1), dilution 1:1000); Myosin IIA (Sigma-Aldrich, catalog# M8064, dilution 1:800); Profilin 1 (Santa Cruz Biotechnology Inc, catalog# sc-137235 (B-10), dilution 1:1000); Profilin 2 (Santa Cruz Biotechnology Inc, catalog# sc-100955 (4K-6), dilution 1:1000); VASP (Santa Cruz Biotechnology Inc, catalog# sc-46668 (A-11), dilution 1:1000); Vinculin (Sigma Aldrich, catalog# V9131, dilution 1:400); IRDye® 680RD Goat anti-Rabbit IgG (LI-COR, Inc., catalog# 926-68071, dilution 1: 5000); IRDye® 800CW Goat anti-Mouse IgG (LI-COR, Inc., catalog# 926-32210, dilution 1:15,000); goat anti-rabbit IgG-HRP (Santa Cruz Biotechnology Inc, catalog# sc-2004, dilution 1:10,000); goat anti-mouse IgG-HRP (Santa Cruz Biotechnology Inc, catalog# sc-2005, dilution 1:10,000); Donkey anti-Mouse IgG (H+L) Highly Cross-Adsorbed Secondary Antibody, Alexa Fluor™ 488 (Invitrogen, catalog# A-21202, dilution 1:500); Donkey anti-Rabbit IgG (H+L) Highly Cross-Adsorbed Secondary Antibody, Alexa Fluor™ 647 (Invitrogen, catalog# A31573, dilution 1:500).

Validation

Antibody validation was provided by manufacture's website (blot or cell images) and/or data provided in the manuscript.

Eukaryotic cell lines

Policy information about cell lines and Sex and Gender in Research

Cell line source(s)

Human foreskin fibroblasts (HFF) were obtained from American Type Culture Collection (catalog no. SCRC-1041).

Authentication

None of the cell line used were authenticated.

Mycoplasma contamination

Cell line routinely (every 3 months) tested negative for mycoplasma contamination using Lonza's MycoAlert PLUS Mycoplasma Detection kit.

Commonly misidentified lines (See ICLAC register)

Cell line used in this study was not found in the list of known misidentified cell lines (ICLAC, Register of cell lines version 11).
